# Supplementary material for: One vaccine to counter many diseases? Modeling the economics of oral polio vaccine against child mortality and COVID-19
Source: Front Public Health. 2022 Oct 5;10:967920. doi: 10.3389/fpubh.2022.967920 (PMC9580701; doi:10.3389/fpubh.2022.967920)
Supplement: Supplementary file 1 [file Data_Sheet_1.docx]

**One vaccine to counter many diseases? Economic evaluation of oral polio vaccine against child mortality and COVID-19**

Angela Y. Chang, Peter Aaby, Michael S. Avidan, Christine S. Benn, Stefano M. Bertozzi, Lawrence Blatt, Konstantin Chumakov, Shabaana A. Khader, Shyam Kottilil, Madhav Nekkar, Mihai G. Netea, Annie Sparrow, Dean T. Jamison

Web appendix

Table of Contents

**Setting 1: Child mortality**

[1.1 Detailed calculation of intervention effectiveness 2](#_Toc74053532)

[1.2 Derivation of campaign costs 3](#_Toc74053533)

[1.3 Derivation of value per statistical life estimates and standard sensitivity analyses for the benefit-cost ratios 4](#_Toc74053534)

[1.4 Sensitivity analyses 5](#_Toc74053535)

**Setting 2: COVID-19**

[2.1 SEIR model description 7](#_Toc74053536)

[2.1.1 Structure and states 7](#_Toc74053537)

[2.1.2 Parameters 9](#_Toc74053538)

[2.1.3 Calibration 10](#_Toc74053539)

[2.2 Modelled pandemic severity in context 13](#_Toc74053540)

[2.3 Vaccine parameters 14](#_Toc74053541)

[2.4 Initial setup and scenario descriptions 15](#_Toc74053542)

[2.5 Derivation of OPV effectiveness against influenza virus infection 16](#_Toc74053543)

[2.6 Derivation of OPV and COVID-19 vaccine delivery costs 17](#_Toc74053544)

[2.7 Full model outputs 18](#_Toc74053545)

[2.7.1 Estimated health outcomes of the baseline and vaccine scenarios 18](#_Toc74053546)

[2.7.2 Full tables of outcomes of interest 21](#_Toc74053547)

[2.8 Derivation of VSL and standard sensitivity analyses for the benefit-cost ratios 24](#_Toc74053548)

[2.9 Sensitivity analyses 27](#_Toc74053549)

[Reference 37](#_Toc74053550)

**Setting 1: Child mortality**

## **Detailed calculation of intervention effectiveness**

Table A1. Estimated effectiveness of OPV campaigns

| Age (month) | Death rate (per 1000)* | Scenario 1: No vaccine | Scenario 2: OPV** |
| --- | --- | --- | --- |
| 0-11 | 50 | 50000 | 45000 [24500-47000] |
| 12-23 | 7 | 6650 | 6150 [5870-6560] |
| 24-35 | 7 | 6600 | 6110 [5830-6510] |
| Total |  | 63250 | 57260 |
| Averted deaths | -- | -- | 5990 [4550-25690]  (6.0 [4.5-25.7] deaths averted per 1000 live births) |
| Estimated campaign effectiveness | -- | -- | 9.5% [7.2-40.6] |

* Death rate among under 5 is 78 per 1000 live births; among 1-4 years is 28 per 1000 children (source: <https://data.worldbank.org/indicator/SH.DYN.MORT?locations=GW>)

** the range reflects the lower and upper estimates of effectiveness

## **Derivation of campaign costs**

No costing study was ever conducted on the OPV campaigns in Guinea Bissau. We therefore relied on cost estimates from the following papers that studied relevant campaigns in similar settings:

Table A2. Summary of relevant campaign costs from the literature

| Study | Country | Program | Cost per recipient (in 2020 USD) * |
| --- | --- | --- | --- |
| Fiedler and Chuko (2008) | Ethiopia | Child Health Days with vitamin A supplementation, de-worming, nutrition screening | $0.72 |
| Fiedler et al. (2014) | Zambia | Child Health Weeks with vitamin A supplementation | $0.76 |
| MOST, USAID (2004) | Ghana, Zambia, Nepal | National vitamin A supplementation campaign | $1.56 |
| Portnoy et al. (2015) | 94 low- and middle-income countries | OPV supplemental immunization activities | $0.77 |
| Cavailler et al. (2006) | Mozambique | Oral cholera vaccine mass vaccination campaign | $1.43 |
| Thompson and Kalkowska (2021) | Low-income countries | OPV in routine immunization of supplemental immunization activities | $0.96 |
| **Average** |  |  | **$1.03** |

* Converted to 2020 USD (U.S. Department of Labor Bureau of Labor Statistic 2021)

## **Derivation of value per statistical life estimates**

The GNI per capita (PPP, international $) in Guinea-Bissau in 2019 was $2230 (World Bank 2021). We calculated the VSL estimates following the standard sensitivity analyses recommended by the benefit-cost analysis reference case, and applied the first set (VSL = $71,000) for the main result (Robinson et al. 2019). Since the study population in this setting is children under age 3, we further explored two age-adjustment scenarios. First, some have suggested that the value placed on reducing risks to children may be lower than those placed on adults (Jamison 2016). To reflect this, we adjusted the main VSL by 50%. In contrast, other studies suggest adjusting the VSL to reflect remaining life expectancy, relative to the median age of the population (Jamison et al. 2013). This is similar to the approach used in cost-effectiveness analysis, which measures changes in the risk of deaths as years of life lost. This yields a VSL that is approximately 1.8 times higher than the normal estimate. All values are reported in 2020 USD.

Table A3. Range of VSL estimates and corresponding benefit-cost ratios (B/C) of OPV campaign for child mortality

|  | Derivation approach | VSL estimate | B/C of OPV campaign |
| --- | --- | --- | --- |
| Standard sensitivity analysis 1 **(main result)** | VSL extrapolated from a US VSL of $9.4 million and US GNI per capita of $57900 (a VSL-to-GNI per capita ratio of 160), using an income elasticity of 1.5. if this approach yields a value of less than 20 times GNI per capita, then 20x GNI per capita should be used instead | 71,000 | 110  [60-590] |
| Standard sensitivity analysis 2 | VSL extrapolated from an OECD VSL-to-GNI per capita ratio of 100 to the target country using an income elasticity of 1.0 | 223,000 | 340  [180-1860] |
| Standard sensitivity analysis 3 | VSL extrapolated from a US VSL-to-GNI per capita ratio of 160 to the target country using an income elasticity of 1. | 357,000 | 550  [290-2980] |
| Age-adjusted (lower) | To reflect the lower value placed on young lives (versus adult lives), we reduce the VSL from the standard sensitivity analysis 1 by 50%. | 36,000 | 50  [30-300] |
| Age-adjusted (higher) | VSL to reflect the differences in life expectancy. VSL from standard sensitivity analysis 1 is multiplied by the ratio of the life expectancy between ages 0 and 35 of the target country. In Guinea-Bissau, the ratio $\frac{e(0)}{e(35)}=\frac{57.8}{32.4}=1.8$ | 127,000 | 200  [100-1060] |

95% uncertainty range in parentheses.

## **Sensitivity analyses**

We conducted one-way sensitivity analyses for the parameters listed in Table A4 and present the tornado graphs on cost-effectiveness and benefit-cost ratios in Figure A1. The lower and upper ranges were chosen based on either the literature or realistic scenarios. The results are the most sensitive to baseline mortality and OPV effectiveness against child mortality. How these two parameters affect the results are presented in Figure 1 of the main text. The size of GNI per capita proportionally affects the VSL, which then affects the benefit-cost ratio. Finally, increasing OPV campaign delivery cost by 50% would lead to approximately 40% increase in the cost-effectiveness ratio and 20% decrease in the benefit-cost ratio.

Table A4. One-way sensitivity analysis parameters

| Parameter | Value in main manuscript | Lower range | Upper range |
| --- | --- | --- | --- |
| Baseline mortality (per 1000 live births) | Year 1: 50  Year 2: 7  Year 3: 7 | Year 1: 10  Year 2: 1.5  Year 3: 1.5 | Year 1: 80  Year 2: 12  Year 3: 12 |
| OPV mortality risk reduction, first dose | 0.90 | 0.94 | 0.49 |
| OPV mortality risk reduction, additional dose | 0.92 | 0.95 | 0.81 |
| OPV vaccine cost | $0.15 | $0.12 | $0.19 |
| OPV campaign delivery cost | $1.03 | $0.72 | $1.56 |
| OPV wastage rate | 10% | 1% | 25% |
| GNI per capita | $2230 | $800 | $12000 |

Figure A1. Tornado graph of one-way sensitivity analyses for child mortality


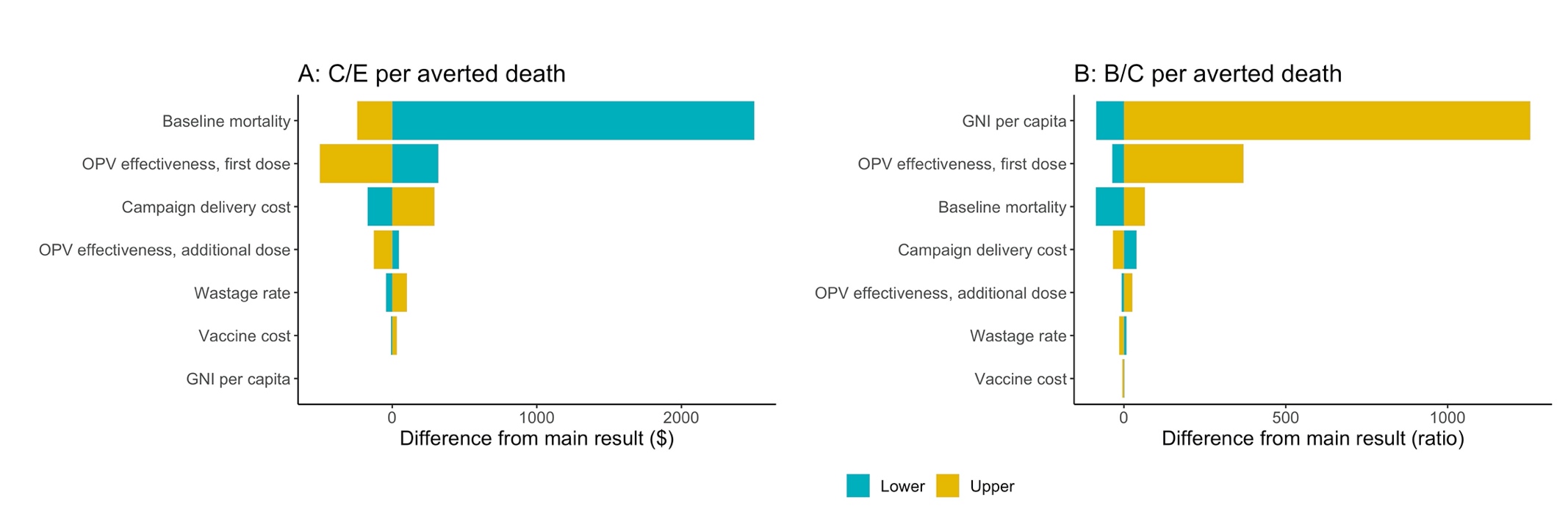


C/E = cost-effectiveness ratio; B/C = benefit-cost ratio

**Setting 2: COVID-19**

## **2.1 SEIR model description**

## **2.1.1 Structure and states**

A dynamic, compartmental Susceptible-Exposed-Infectious-Recovered (SEIR) model was developed to estimate the population benefits of different vaccine scenarios. See Figure A2 for the model diagram. We defined a total of 20 model states:

1. S: susceptible
2. E: exposed
3. Iasymp: asymptomatic and infectious
4. Isymp: symptomatic and infectious
5. Hosp: requiring hospitalization due to COVID-19
6. Icu: requiring intensive care due to COVID-19
7. S_vb: susceptible, vaccinated with OPV in full effectiveness
8. E_vb: exposed, vaccinated with OPV in full effectiveness
9. Iasymp_vb: asymptomatic and infectious, vaccinated with OPV in full effectiveness
10. Isymp_vb: symptomatic and infectious, vaccinated with OPV in full effectiveness
11. Hosp_vb: requiring hospitalization due to COVID-19, vaccinated with OPV in full effectiveness
12. Icu_vb: requiring intensive care due to COVID-19, vaccinated with OPV in full effectiveness
13. S_v1: susceptible, vaccinated with the COVID-19 vaccine in full effectiveness
14. E_v1: exposed, vaccinated with the COVID-19 vaccine in full effectiveness
15. Iasymp_v1: asymptomatic and infectious, vaccinated with the COVID-19 vaccine in full effectiveness
16. Isymp_v1: symptomatic and infectious, vaccinated with the COVID-19 vaccine in full effectiveness
17. Hosp_v1: requiring hospitalization due to COVID-19, vaccinated with the COVID-19 vaccine in full effectiveness
18. Icu_v1: requiring intensive care due to COVID-19, vaccinated with the COVID-19 vaccine in full effectiveness
19. Recovered (not included in Figure A2): recovered, non-infectious
20. Dead (not included in Figure A2)

Individuals in states Iasymp, Isymp, Iasymp_vb, Isymp_vb, Iasymp_v1, Isymp_v1 are infectious. Individuals in states S, S_vb, and S_v1 are susceptible to infection.

In Figure A2, the first row represents the disease transition of individuals who are not vaccinated. Once exposed, the individual will move from being exposed, infectious and asymptomatic, infectious and symptomatic, hospitalized, and requiring intensive care, based on transition rates. When the COVID-19 vaccine is available, individuals who are expected to receive the vaccine will move to the third row at a rate determined by the days required after receiving the vaccine for it to be fully effective (Panel A). When OPV is co-administered, individuals who are expected to receive the vaccines will first move to the second row, and then transition to the third row at a rate that depends on the delays related to receiving the COVID-19 vaccine (Panel B).

Figure A2. Model diagram

1. With the COVID-19 vaccine and no OPV

1. Co-administration of the COVID-19 vaccine and OPV

## **2.1.2 Parameters**

Table A5. Model parameters and definitions

| **Parameter** | **Definition** |
| --- | --- |
| Time step | 1 day |
| Transition rate, $\delta$ | $\delta_{e}$: transition rate from E to Iasymp  $\delta_{a}$: transition rate from Iasymp to Isymp  $\delta_{h}$: transition rate from Isymp to Hosp  With vaccines: each parameter is modified by OPV and the COVID-19 vaccine effectiveness against disease severity. For example,  $\delta_{e\_vb}=\delta_{e}*\left( 1-{\mathrm{vx}_{\mathrm{eff}_{\mathrm{sev}}}}_{\mathrm{OPV}} \right); \delta_{e\_v1}=\delta_{e}*(1-{vx\_eff\_sev}_{\mathrm{COVID}})$ |
| Recovery rate, $\gamma$ | $\gamma_{a}$: recovery rate from Iasymp  $\gamma_{s}$: recovery rate from Isymp  $\gamma_{h}$: recovery rate from Hosp  $\gamma_{i}$: recovery rate from Icu  With vaccines: each parameter is modified by OPV and the COVID-19 vaccine effectiveness against disease severity. For example,  $\gamma_{a\_vb}=\gamma_{a}*\left( 1+{\mathrm{vx}_{\mathrm{eff}_{\mathrm{sev}}}}_{\mathrm{OPV}} \right); \gamma_{a\_v1}=\gamma_{a}*(1+{vx\_eff\_sev}_{\mathrm{COVID}})$ |
| COVID-19 specific death rate, $\mu$ | $\mu_{s}$: death rate at Isymp  $\mu_{h}$: death rate at Hosp  $\mu_{i}$: death rate at Icu  With vaccines: each parameter is modified by OPV and COVID-19 vaccine effectiveness against disease severity. For example,  $\mu_{s\_vb}=\mu_{b}*\left( 1-{\mathrm{vx}_{\mathrm{eff}_{\mathrm{sev}}}}_{\mathrm{OPV}} \right); \mu_{s\_v1}=\mu_{b}*(1-{vx\_eff\_sev}_{\mathrm{COVID}})$ |
| Infectiousness, $\beta$ | $\beta$ = $\beta_{\mathrm{asymp}}$ = $\beta_{\mathrm{symp}}$ = $R_{0}*\frac{\delta_{a}+\gamma_{a}}{(1+\frac{\delta_{a}}{\delta_{s}+\gamma_{s}+\mu_{s}})}$  $\beta'$ is the weighted sum of ${\beta,\beta}_{\mathrm{vb}}$, $\beta_{v1}$, weighed by the size of infectious populations of each state. i.e.,  Weighted force of infection at time t:  $\beta^{'}\left( t \right)= \beta_{\mathrm{asymp}}*Iasymp\left( t \right)+ \beta_{\mathrm{symp}}*Isymp\left( t \right)+\beta_{\mathrm{asym}p_{\mathrm{vb}}}*Iasymp_{\mathrm{vb}\left( t \right)}+ \beta_{\mathrm{sym}p_{\mathrm{vb}}}*Isymp_{\mathrm{vb}\left( t \right)}+ \beta_{\mathrm{asym}p_{v1}}*Iasymp_{v1\left( t \right)}+ \beta_{\mathrm{sym}p_{v1}}*Isymp_{v1\left( t \right)}$  We assume the ratio of the infectiousness between asymptomatic and symptomatic infections at 0.75.  With vaccines: $\beta_{\mathrm{vb}}$ and $\beta_{v1}$ are modified by OPV and the COVID-19 vaccine effectiveness against infectivity:  $\beta_{\mathrm{vb}}=\beta*(1-{vx\_eff\_inf}_{\mathrm{OPV}})$  $\beta_{v1}=\beta*(1-{vx\_eff\_inf}_{\mathrm{COVID}})$ |
| Vaccination rate, $v$ | $v_{b}$: the rate in which OPV is administered and becomes fully effective  $v_{1}$: the rate in which the COVID-19 vaccine is administered and becomes fully effective. This reflects the delays due to development and trials, manufacturing, procurement, and administration of the vaccine, as well as the days it takes post administration for the vaccine to be fully effective |

## **2.1.3 Calibration**

The first wave of the COVID-19 epidemic among adults in India was selected to illustrate the potential use of OPV during the pandemic. India was chosen because it has rigorous seroprevalence data from three rounds of national seroprevalence surveys, and faced delays in immunizing a large proportion of the population with the COVID-19 vaccine, reflecting the situation that are faced by other lower income countries. For model calibration, we carefully followed the estimates reported by the national surveys (Murhekar et al. 2021; Murhekar et al. 2021; Murhekar et al. 2020). We set the first day of the simulation to May 11, 2020, when 0.7% of the population was estimated to be sero-positive. We assumed that 25% of this population was still infectious. In other words, 0.175% was set as the initial exposed population and the remaining 0.525% set as the recovered population. At the end of the third seroprevalence study, 24.3% of adults were estimated to be positive, and our model was calibrated so that similar levels of total infections would be reached by approximately 250 days. An estimated 3.7% of all infections were reported in India, all of which we assume were symptomatic. We further assumed that only 50% of symptomatic cases were reported and calibrated the model so that approximately 7.4% (twice the size of the actual reported) were symptomatic cases, and the remaining 92.6% remained asymptomatic, consistent with epidemiological surveys in Karnataka and Andhra Pradesh and at the national level (Bedi 2020; Kumar et al. 2021; Murhekar et al. 2021). Reported infection-fatality rate (IFR) was 0.094-0.107% at the end of the second survey (98-134 days since May 11, 2020). Finally, the peak of the daily reported case occurred mid-September, 128 days since our start date. Estimated R0 based on these parameters is 1.15.

Table A6. Model calibration targets and model outputs

| Macro parameter | Target | Model output |
| --- | --- | --- |
| Ever infected (%) by day 242 (Jan 8, 2021) | 20-23% adult seroprevalence between Dec 17, 2020-Jan 8, 2021 (from the third prevalence study) | 23.0% |
| % symptomatic among infected | 3.2-3.8% were reported  (from the second prevalence study, approximately 1 in 26-31 cases)  3.7% reported (1 in 27 cases, third prevalence study) | Assuming 50% of symptomatic cases were reported, then % symptomatic among infected would be 3.7 *2 = 7.4%  Current model output: 7.45% |
| Peak daily reported case | ~ mid-September 2020  (~128 days) | 138 days |
| Infection-fatality rate (IFR) at day 242 | 0.0943-0.1065%  (second seroprevalence study) | 0.10% |

Table A7. Calibrated input data and assumptions

|  | Value |
| --- | --- |
| Average days spent in state (day) |  |
| Exposed | 3 |
| Asymptomatic | 3 |
| Symptomatic | 4 |
| Hospitalized | 4 |
| ICU | 10 |
| Transition proportion (%) |  |
| Exposed to asymptomatic | 100 |
| Asymptomatic to symptomatic | 7.4 |
| Symptomatic to hospitalized | 7.5 |
| Hospitalized to ICU | 10 |
| Mortality proportion (%) |  |
| Exposed | 0 |
| Asymptomatic | 0 |
| Symptomatic | 0.8 |
| Hospitalized | 7 |
| ICU | 15 |
| Basic reproduction number R0 | 1.15 |

Figure A3. Modelled baseline disease dynamic

The green area at the bottom of panel A is magnified in panel B


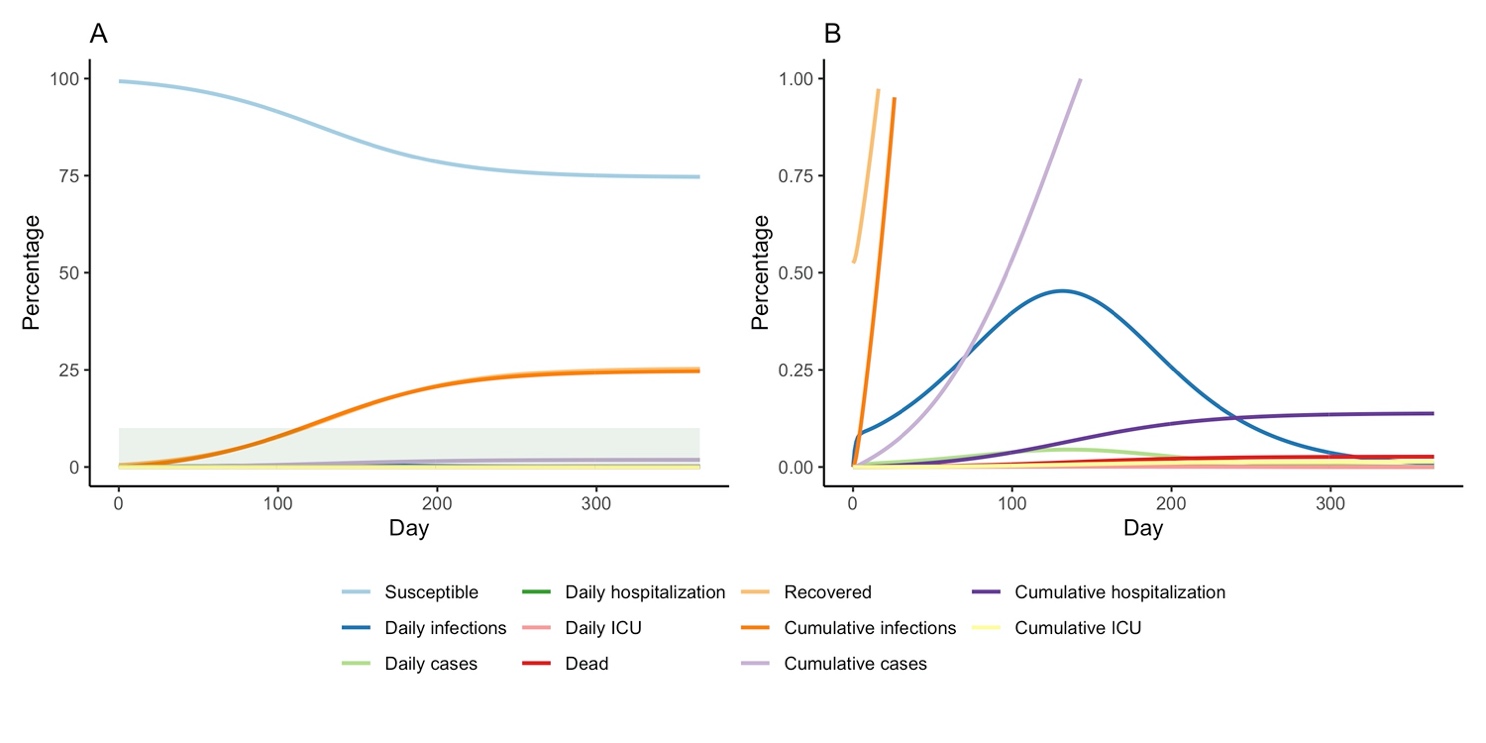


Figure A4. Modelled baseline disease dynamic: cumulative and daily infections (asymptomatic) and cases (symptomatic)


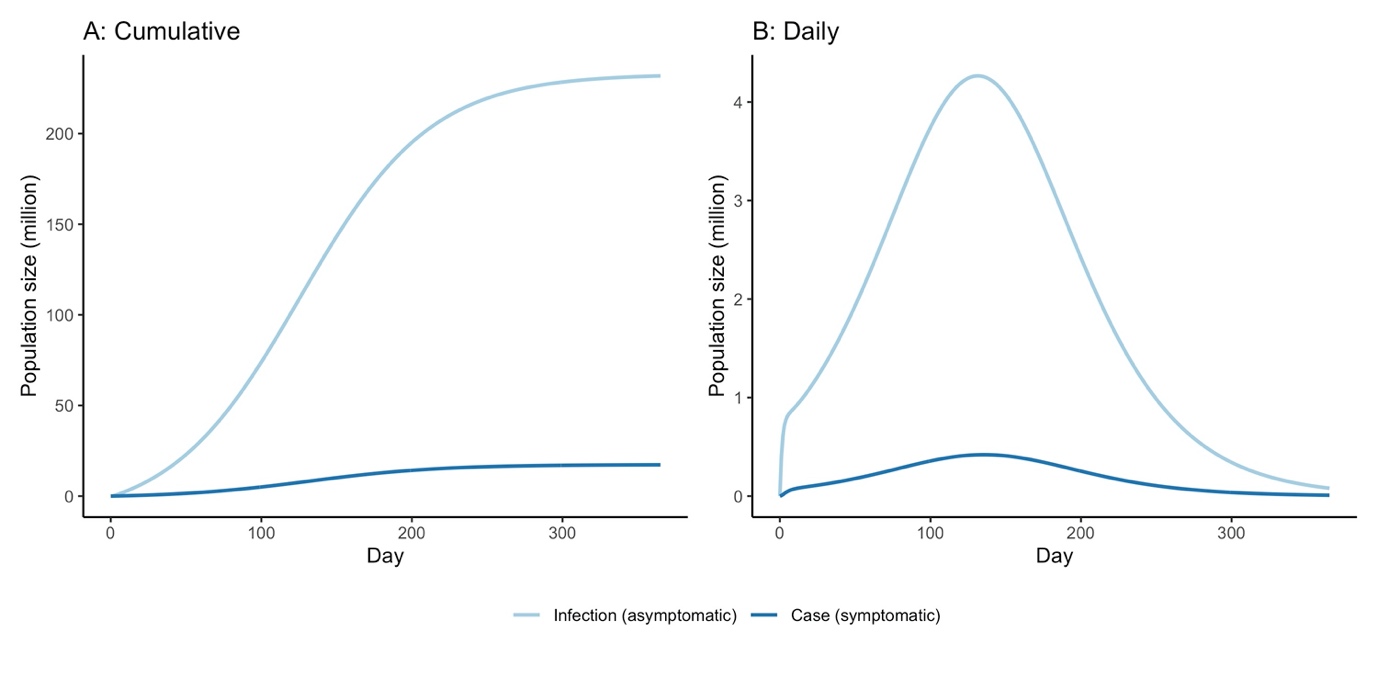


## **2.2 Modelled pandemic severity in context**

The number of events that an immunization program can avert depends directly on the incidence rate of new infections. Our analysis used India’s first wave as the basis for modeling and calculations. Peak incidence (near September 16, 2020) was about 68 new cases per million population, as reported in Our World in Data (Roser et al. 2020). The table shows a range of other peak wave incidence rates, most of which are noticeably higher than 68. For that reason, our model’s estimates of cost per death averted and other outcome parameters are (deliberately) conservative.

Two detailed but important points should be made. First, in our model we further adjust the epidemic based on India’s national seroprevalence studies, such that the daily new reported cases per million people is twice (~112) of what is reported from Our World in Data. However, even with this higher estimate, India’s first wave is relatively milder than many other waves observed around the world. Second, country comparisons of reported cases are difficult because the differences reflect variability in testing capabilities, reporting systems, definition of cases, among other factors, in addition to differences in the epidemics (such as ratio of asymptomatic to symptomatic cases).

Table A8. Modelled pandemic severity in context

| Country / wave | Date of peak | Daily new confirmed cases per million people at peak* |
| --- | --- | --- |
| India / wave 1  (modelled in this paper) | Sep 16, 2020 | 68 |
| India / wave 2 | May 9, 2021 | 283 |
| World / wave 1 | Jan 11, 2021 | 95 |
| Brazil / wave 1 | July 29, 2020 | 218 |
| Brazil / wave 2 | Mar 27, 2021 | 363 |
| South Korea / wave 3 | Dec 25, 2020 | 20 |
| Spain / wave 1 | Mar 31, 2020 | 171 |
| Spain / wave 3 | Jan 26, 2021 | 792 |

* 7-day moving averages are shown.

Data source: Our World in Data (Roser et al. 2020)

## **2.3 Vaccine parameters**

Table A9. Vaccine parameters

|  | COVID-19 vaccine | OPV |
| --- | --- | --- |
| Vaccine coverage | 30 and 50% | 30 and 50% |
| Effectiveness against infectivity among people who are infected | 74% [65-95] (FDA 2021) | 0 |
| Effectiveness against infection (susceptibility) | 74% [65-95] (FDA 2021) | 20% [0-64] |
| Effectiveness against disease severity | 95% [75-99] | 20% [0-64] |
| Number of days from administration until fully effective | 28 days [7-35] (FDA 2021) | 1 day |

## **2.4 Initial setup and scenario descriptions**

We assume a population size N = 1 so that the results can be interpreted as proportions. We set the initial number of people exposed (in state E) at 0.00175 (or 0.175% of the whole population), corresponding to the proportion of people who were sero-positive in the first Indian seroprevalence study (Murhekar et al. 2021). We set 0.525% of the population to be in the recovered state, and the remaining population started in the susceptible state (S). In the baseline scenario, we assumed a vaccine coverage of 0%.

Two intervention scenarios with coadministration of the COVID-19 vaccine and OPV were explored. In both scenarios, the COVID-19 vaccine is introduced with an implementation delay to reflect the time required to develop, test, approve, manufacture, procure, and administer. It also has a lag time between when the vaccine is administered and when it achieves full effect (effectiveness delay). For both scenarios, the comparison is made against scenarios without OPV (i.e., only the COVID-19 vaccine).

In the simultaneous administration scenario, both the COVID-19 vaccine and OPV are administered on the same day. Since we assumed OPV’s effectiveness against COVID-19 to start nearly immediately, it will provide some protection against the disease during the COVID-19 vaccine’s effectiveness delay. More specifically, in the main result, vaccine recipients (30%) will first move from S to S_vb (bridge state) on the day and benefit from OPV, and then at a transition rate of 1/28 (28 days until fully effective) move from S_vb to S_v1.

In the COVID-19 vaccine delay scenario, we assumed a longer implementation delay for the COVID-19 vaccine, and OPV is administered *d* days before the availability of the COVID-19 vaccine. In other words, these individuals will first receive OPV and then later receive the COVID-19 vaccine. In the main result, vaccine recipients will first move from S to S_vb on the day in which OPV is administered first and stay in the bridge states until the COVID-19 vaccine becomes available. Then, they will face a short delay due to COVID-19 vaccine’s effectiveness delay. In the main result, approximately 50% of the vaccinated population will reach full effectiveness by 14 days (half of the delayed days) and 100% of the vaccinated population will reach full effectiveness by 28 days.

## **2.5 Derivation of OPV effectiveness against influenza virus infection**

We estimate the effect of OPV against influenza and acute respiratory infections from the study conducted by Chumakov et al. (1992). Specifically, we converted the estimates reported in their Table 1 (columns A-C below) to vaccine effectiveness, presented as percentage reduction in incidence (column E). However, we also noted that other studies suggested effectiveness below 30% (Fisker and Bandim Health Project 2020; Seppälä et al. 2011). In the main analysis, we apply 20% as OPV’s effectiveness against COVID-19’s susceptibility and severity for the headline results.

Table A10. OPV effectiveness against influenza and acute respiratory infections

Calculations based on Table 1 of Chumakov et al. (1992)

|  | A | B | C | D | E |
| --- | --- | --- | --- | --- | --- |
| Study | Study population size (%) | % disease cases in control group | % disease cases in treatment group | Incidence reduction, fold (A) | Estimated vaccine effectiveness in reducing incidence* |
| 1 | 10544 (12%) | 26.41 | 5.81 | 4.5 | 0.78 |
| 2 | 59558 (69%) | 28.9 | 15.5 | 1.9 | 0.47 |
| 3 | 4819 (6%) | 20.2 | 7.0 | 2.9 | 0.66 |
| 4 | 11077 (13%) | 64.9 | 11.2 | 5.8 | 0.83 |
| Summary | 85998 | -- | -- | 3.8 (reported)  2.8 (weighted average**) | 0.64 (weighted average**) |

* vaccine effectiveness = $1-\frac{1}{incidence reduction (col D)}$

**weighted by the study population size (col A)

## **2.6 Derivation of OPV and COVID-19 vaccine delivery costs**

OPV: There is no existing study that provides an estimate of the OPV delivery cost in India. We therefore relied on cost estimates from the following papers that studied relevant campaigns in similar settings:

Table A11. Summary of relevant vaccine delivery costs

| Study | Country | Program | Cost per beneficiary (in 2020 USD)* |
| --- | --- | --- | --- |
| Kar et al. (2014) | India | Oral cholera vaccine mass vaccination campaign | $0.56 |
| MOST, USAID (2004) | Ghana, Zambia, Nepal | National vitamin A supplementation campaign | $1.56 |
| Portnoy et al. (2015) | 94 low- and middle-income countries | OPV supplemental immunization activities | $0.77 |
| Thompson and Kalkowska (2021) | Low-income countries | OPV in routine immunization of supplemental immunization activities | $0.96 |
| **Average** |  |  | **$0.96** |

* Converted to 2020 USD (U.S. Department of Labor Bureau of Labor Statistic 2021)

COVID-19 vaccine: Cost of delivering COVID-19 vaccine in India was estimated by a group of experts from the World Health Organization, UNICEF, and Gavi the Vaccine Alliance at $1.07 per dose supplied (2020 USD) (Griffiths et al. 2021). This estimate includes costs associated with planning and coordination, training, cold chain equipment, pharmacovigilance, vaccine certificates, personal protective equipment, transport, waste management, and outreach costs. It does not include costs associated with human resources. We therefore add an additional $0.42 [$0.10-1.17] of labor cost per dose estimated for India (2020 USD) (Portnoy et al. 2020). The total delivery cost for COVID-19 vaccine in India is thus $1.49.

## **2.7 Full model outputs**

## **2.7.1 Estimated health outcomes of the baseline and vaccine scenarios**

**Table A12. Modelled health outcomes for COVID-19 vaccine only scenarios**

| Days into wave vaccines administered, *t* (day) | Ultimate number of cumulative infections (%) | Ultimate number of cumulative symptomatic cases (%) | Ultimate number of cumulative hospitalizations (%) | Ultimate number of cumulative ICUs (%) | Ultimate number of cumulative deaths (%) |
| --- | --- | --- | --- | --- | --- |
| Baseline  (no vaccine) | 25 | 1.8 | 0.1 | 0.01 | 0.03 |
| 25 | 3.9 | 0.3 | 0.02 | 0.00 | 0.00 |
| 50 | 6.5 | 0.5 | 0.04 | 0.00 | 0.01 |
| 100 | 13.1 | 1.0 | 0.07 | 0.01 | 0.01 |
| 200 | 22.5 | 1.7 | 0.13 | 0.01 | 0.02 |
| 300 | 24.5 | 1.8 | 0.14 | 0.01 | 0.03 |

**Table A13. Modelled health outcomes for baseline (no vaccination) and two vaccine scenarios**

For both scenarios, we present the health outcomes for two vaccine coverage rates (30 and 50%) and three OPV effectiveness (0, 20, 60%).

Simultaneous administration scenario

| Vaccine coverage | OPV effectiveness, *e* (%) | Days into wave vaccines administered, *t* (day) | Ultimate number of cumulative infections (%) | Ultimate number of cumulative symptomatic cases (%) | Ultimate number of cumulative hospitalizations (%) | Ultimate number of cumulative ICUs (%) | Ultimate number of cumulative deaths (%) | Infection-fatality rate | Case-fatality rate |
| --- | --- | --- | --- | --- | --- | --- | --- | --- | --- |
| -- | -- | Baseline  (no vaccine) | 25 | 1.8 | 0.1 | 0.01 | 0.03 | 0.11 | 1.44 |
| 30% | 0% | 25 | 25 | 1.8 | 0.1 | 0.01 | 0.03 | 0.11 | 1.44 |
| 30% | 0% | 50 | 25 | 1.8 | 0.1 | 0.01 | 0.03 | 0.11 | 1.44 |
| 30% | 0% | 100 | 25 | 1.8 | 0.1 | 0.01 | 0.03 | 0.11 | 1.44 |
| 30% | 0% | 200 | 25 | 1.8 | 0.1 | 0.01 | 0.03 | 0.11 | 1.44 |
| 30% | 0% | 300 | 25 | 1.8 | 0.1 | 0.01 | 0.03 | 0.11 | 1.44 |
| 30% | 20% | 25 | 3.1 | 0.2 | 0.02 | 0.002 | 0.003 | 0.1 | 1.4 |
| 30% | 20% | 50 | 5.5 | 0.4 | 0.03 | 0.003 | 0.006 | 0.1 | 1.41 |
| 30% | 20% | 100 | 11.9 | 0.9 | 0.06 | 0.006 | 0.012 | 0.1 | 1.42 |
| 30% | 20% | 200 | 22.1 | 1.6 | 0.12 | 0.012 | 0.023 | 0.11 | 1.43 |
| 30% | 20% | 300 | 24.4 | 1.8 | 0.14 | 0.014 | 0.026 | 0.11 | 1.44 |
| 30% | 60% | 25 | 2.4 | 0.2 | 0.01 | 0.001 | 0.003 | 0.11 | 1.42 |
| 30% | 60% | 50 | 4.5 | 0.3 | 0.02 | 0.002 | 0.005 | 0.1 | 1.43 |
| 30% | 60% | 100 | 10.7 | 0.8 | 0.06 | 0.006 | 0.011 | 0.1 | 1.43 |
| 30% | 60% | 200 | 21.7 | 1.6 | 0.12 | 0.012 | 0.023 | 0.11 | 1.44 |
| 30% | 60% | 300 | 24.4 | 1.8 | 0.14 | 0.014 | 0.026 | 0.11 | 1.44 |
| 50% | 0% | 25 | 25 | 1.8 | 0.1 | 0.01 | 0.03 | 0.11 | 1.44 |
| 50% | 0% | 50 | 25 | 1.8 | 0.1 | 0.01 | 0.03 | 0.11 | 1.44 |
| 50% | 0% | 100 | 25 | 1.8 | 0.1 | 0.01 | 0.03 | 0.11 | 1.44 |
| 50% | 0% | 200 | 25 | 1.8 | 0.1 | 0.01 | 0.03 | 0.11 | 1.44 |
| 50% | 0% | 300 | 25 | 1.8 | 0.1 | 0.01 | 0.03 | 0.11 | 1.44 |
| 50% | 20% | 25 | 2.1 | 0.2 | 0.01 | 0.001 | 0.002 | 0.1 | 1.37 |
| 50% | 20% | 50 | 4.1 | 0.3 | 0.02 | 0.002 | 0.004 | 0.1 | 1.39 |
| 50% | 20% | 100 | 10.4 | 0.7 | 0.06 | 0.005 | 0.011 | 0.1 | 1.4 |
| 50% | 20% | 200 | 21.7 | 1.6 | 0.12 | 0.012 | 0.023 | 0.11 | 1.43 |
| 50% | 20% | 300 | 24.4 | 1.8 | 0.14 | 0.014 | 0.026 | 0.11 | 1.44 |
| 50% | 60% | 25 | 1.6 | 0.1 | 0.01 | 0.001 | 0.002 | 0.11 | 1.41 |
| 50% | 60% | 50 | 3.4 | 0.3 | 0.02 | 0.002 | 0.004 | 0.1 | 1.42 |
| 50% | 60% | 100 | 9.4 | 0.7 | 0.05 | 0.005 | 0.01 | 0.1 | 1.43 |
| 50% | 60% | 200 | 21.3 | 1.6 | 0.12 | 0.012 | 0.023 | 0.11 | 1.44 |
| 50% | 60% | 300 | 24.3 | 1.8 | 0.14 | 0.014 | 0.026 | 0.11 | 1.44 |

Delayed availability of COVID-19 vaccine scenario

Days into wave that OPV is administered (*t*) = 50 days

| Vaccine coverage | OPV effectiveness (e) | Delay of COVID-19 vaccine (*d*) | Ultimate number of cumulative infections | Ultimate number of cumulative symptomatic cases | Ultimate number of cumulative hospitalizations | Ultimate number of cumulative ICUs | Ultimate number of cumulative deaths | Infection-fatality rate | Case-fatality rate |
| --- | --- | --- | --- | --- | --- | --- | --- | --- | --- |
| -- | -- | Baseline | 25 | 1.8 | 0.1 | 0.01 | 0.03 | 0.11 | 1.44 |
| 30% | 0% | 50 days | 25 | 1.8 | 0.1 | 0.01 | 0.03 | 0.11 | 1.44 |
| 30% | 0% | 100 days | 25 | 1.8 | 0.1 | 0.01 | 0.03 | 0.11 | 1.44 |
| 30% | 0% | 150 days | 25 | 1.8 | 0.1 | 0.01 | 0.03 | 0.11 | 1.44 |
| 30% | 20% | 50 days | 8 | 0.6 | 0.04 | 0.004 | 0.008 | 0.1 | 1.37 |
| 30% | 20% | 100 days | 9.6 | 0.7 | 0.05 | 0.005 | 0.009 | 0.1 | 1.36 |
| 30% | 20% | 150 days | 10.5 | 0.7 | 0.05 | 0.005 | 0.01 | 0.1 | 1.35 |
| 30% | 60% | 50 days | 4.9 | 0.4 | 0.03 | 0.003 | 0.005 | 0.1 | 1.42 |
| 30% | 60% | 100 days | 5 | 0.4 | 0.03 | 0.003 | 0.005 | 0.1 | 1.41 |
| 30% | 60% | 150 days | 5 | 0.4 | 0.03 | 0.003 | 0.005 | 0.1 | 1.41 |
| 50% | 0% | 50 days | 25 | 1.8 | 0.1 | 0.01 | 0.03 | 0.11 | 1.44 |
| 50% | 0% | 100 days | 25 | 1.8 | 0.1 | 0.01 | 0.03 | 0.11 | 1.44 |
| 50% | 0% | 150 days | 25 | 1.8 | 0.1 | 0.01 | 0.03 | 0.11 | 1.44 |
| 50% | 20% | 50 days | 5.7 | 0.4 | 0.03 | 0.003 | 0.005 | 0.09 | 1.33 |
| 50% | 20% | 100 days | 6.4 | 0.4 | 0.03 | 0.003 | 0.006 | 0.09 | 1.32 |
| 50% | 20% | 150 days | 6.7 | 0.5 | 0.03 | 0.003 | 0.006 | 0.09 | 1.31 |
| 50% | 60% | 50 days | 3.6 | 0.3 | 0.02 | 0.002 | 0.004 | 0.1 | 1.41 |
| 50% | 60% | 100 days | 3.6 | 0.3 | 0.02 | 0.002 | 0.004 | 0.1 | 1.41 |
| 50% | 60% | 150 days | 3.6 | 0.3 | 0.02 | 0.002 | 0.004 | 0.1 | 1.41 |

Days into wave that OPV is administered (*t*) = 100 days

| Vaccine coverage | OPV effectiveness (e) | Delay of COVID-19 vaccine (*d*) | Ultimate number of cumulative infections | Ultimate number of cumulative symptomatic cases | Ultimate number of cumulative hospitalizations | Ultimate number of cumulative ICUs | Ultimate number of cumulative deaths | Infection-fatality rate | Case-fatality rate |
| --- | --- | --- | --- | --- | --- | --- | --- | --- | --- |
| -- | -- | Baseline | 25 | 1.8 | 0.1 | 0.01 | 0.03 | 0.11 | 1.44 |
| 30% | 0% | 50 days | 25 | 1.8 | 0.1 | 0.01 | 0.03 | 0.11 | 1.44 |
| 30% | 0% | 100 days | 25 | 1.8 | 0.1 | 0.01 | 0.03 | 0.11 | 1.44 |
| 30% | 0% | 150 days | 25 | 1.8 | 0.1 | 0.01 | 0.03 | 0.11 | 1.44 |
| 30% | 20% | 50 days | 14.3 | 1 | 0.08 | 0.007 | 0.014 | 0.1 | 1.39 |
| 30% | 20% | 100 days | 15.2 | 1.1 | 0.08 | 0.008 | 0.015 | 0.1 | 1.38 |
| 30% | 20% | 150 days | 15.5 | 1.1 | 0.08 | 0.008 | 0.015 | 0.1 | 1.38 |
| 30% | 60% | 50 days | 11.1 | 0.8 | 0.06 | 0.006 | 0.011 | 0.1 | 1.42 |
| 30% | 60% | 100 days | 11.2 | 0.8 | 0.06 | 0.006 | 0.012 | 0.1 | 1.42 |
| 30% | 60% | 150 days | 11.2 | 0.8 | 0.06 | 0.006 | 0.012 | 0.1 | 1.42 |
| 50% | 0% | 50 days | 25 | 1.8 | 0.1 | 0.01 | 0.03 | 0.11 | 1.44 |
| 50% | 0% | 100 days | 25 | 1.8 | 0.1 | 0.01 | 0.03 | 0.11 | 1.44 |
| 50% | 0% | 150 days | 25 | 1.8 | 0.1 | 0.01 | 0.03 | 0.11 | 1.44 |
| 50% | 20% | 50 days | 12.2 | 0.9 | 0.06 | 0.006 | 0.012 | 0.1 | 1.37 |
| 50% | 20% | 100 days | 12.6 | 0.9 | 0.06 | 0.006 | 0.012 | 0.1 | 1.36 |
| 50% | 20% | 150 days | 12.7 | 0.9 | 0.06 | 0.006 | 0.012 | 0.1 | 1.36 |
| 50% | 60% | 50 days | 9.5 | 0.7 | 0.05 | 0.005 | 0.01 | 0.1 | 1.42 |
| 50% | 60% | 100 days | 9.5 | 0.7 | 0.05 | 0.005 | 0.01 | 0.1 | 1.42 |
| 50% | 60% | 150 days | 9.5 | 0.7 | 0.05 | 0.005 | 0.01 | 0.1 | 1.42 |

## **2.7.2 Full tables of outcomes of interest (deaths averted per thousand immunized, cost-effectiveness and benefit-cost ratios)**

**Table A14. Outcomes of COVID-19 vaccine + OPV schedule, with 95% uncertainty range**

Note that the first table is the same as in the main manuscript but with 95% uncertainty ranges in the parentheses.

**Vaccine coverage = 30%**

Simultaneous administration scenario

|  | Outcome | | | | | |
| --- | --- | --- | --- | --- | --- | --- |
|  | e = 20% | | | e = 60% | | |
| Days into wave* vaccines administered (*t*) | DATI | C/E (in thousands of US$) | B/C | DATI | C/E (in thousands of US$) | B/C |
| 25 | 0.03 [0.005-0.1] | 40 [7-280] | 10 [1-50] | 0.05 [0.009-0.2] | 23 [4-150] | 17 [3-90] |
| 50 | 0.04 [0.006-0.2] | 30 [5-200] | 13 [2-70] | 0.07 [0.01-0.3] | 17 [3-110] | 22 [4-130] |
| 100 | 0.05 [0.008-0.1] | 23 [6-150] | 17 [3-60] | 0.09 [0.02-0.2] | 14 [4-80] | 28 [5-100] |
| 200 | 0.02 [0.002-0.02] | 65 [35-890] | 6 [0.4-10] | 0.03 [0.002-0.03] | 41 [22-560] | 10 [1-20] |
| 300 | 0.002 [0-0.005] | 540 [160-59300] | 0.7 [0.01-2] | 0.004 [0-0.008] | 340 [100-37700] | 1.1 [0.01-4] |

Note: if e = 0%, costs outweigh benefits and should not be considered.

Delayed availability of COVID-19 vaccine scenario

|  | Days into wave* that OPV is administered (*t*) | | | | | | | | | | | |
| --- | --- | --- | --- | --- | --- | --- | --- | --- | --- | --- | --- | --- |
|  | *t* = 50 | | | | | | *t* = 100 | | | | | |
|  | e = 20% | | | e = 60% | | | e = 20% | | | e = 60% | | |
| Delay of COVID-19 vaccine (*d*) | DATI | C/E (thousands of US$) | B/C* | DATI | C/E (thousands of US$) | B/C | DATI | C/E (thousands of US$) | B/C* | DATI | C/E (thousands of US$) | B/C* |
| 50 days | 0.2  [0.1-0.5] | 6.1  [1.6-17] | 60  [40-60] | 0.3  [0.1-0.8] | 4.2  [1-12] | 90  [60-90] | 0.2  [0.1-0.3] | 6.1  [2.5-16] | 60  [40-60] | 0.3  [0.1-0.4] | 4.2  [1.7-11] | 90  [50-90] |
| 100 days | 0.4  [0.2-0.6] | 3.3  [1.2-9] | 120  [40-120] | 0.5  [0.3-1] | 2.4  [0.7-7] | 160  [60-160] | 0.3  [0.2-0.4] | 4.1  [2-10] | 100  [40-100] | 0.4  [0.3-0.5] | 2.9  [1.4-7] | 130  [50-130] |
| 150 days | 0.5  [0.3-0.6] | 2.6  [1.2-6] | 150  [60-150] | 0.6  [0.4-1] | 1.9  [0.7-5] | 200  [80-200] | 0.3  [0.3-0.4] | 3.5  [1.9-8] | 110  [50-110] | 0.5  [0.3-0.5] | 2.6  [1.3-6] | 150  [60-150] |

Note: if e = 0%, costs outweigh benefits and should not be considered.

**Vaccine coverage = 50%**

Simultaneous administration scenario

|  | Outcome | | | | | |
| --- | --- | --- | --- | --- | --- | --- |
|  | e = 20% | | | e = 60% | | |
| Days into wave* vaccines administered (*t*) | DATI | C/E (in thousands of US$) | B/C | DATI | C/E (in thousands of US$) | B/C |
| 25 | 0.02 [0.003-0.1] | 79 [16-500] | 5 [1-20] | 0.02 [0.005-0.1] | 53 [11-300] | 7 [1-40] |
| 50 | 0.02 [0.004-0.1] | 55 [9-340] | 7 [1-40] | 0.03 [0.01-0.1] | 37 [6-200] | 11 [2-60] |
| 100 | 0.03 [0.005-0.1] | 38 [9-230] | 10 [2-40] | 0.05 [0.01-0.1] | 25 [6-140] | 15 [3-60] |
| 200 | 0.01 [0.001-0.02] | 93 [51-1140] | 4 [0.3-10] | 0.02 [0.002-0.02] | 65 [36-790] | 6 [0.5-10] |
| 300 | 0.002 [0-0.004] | 740 [220-75900] | 0.5 [0.01-2] | 0.002 [0-0.005] | 520 [150-52800] | 0.8 [0.01-3] |

Note: if e = 0%, costs outweighs benefits and should not be considered.

Delayed availability of COVID-19 vaccine scenario

|  | Days into wave* that OPV is administered (*t*) | | | | | | | | | | | |
| --- | --- | --- | --- | --- | --- | --- | --- | --- | --- | --- | --- | --- |
|  | *t* = 50 | | | | | | *t* = 100 | | | | | |
|  | e = 20% | | | e = 60% | | | e = 20% | | | e = 60% | | |
| Delay of COVID-19 vaccine (*d*) | DATI | C/E (thousands of US$) | B/C* | DATI | C/E (thousands of US$) | B/C | DATI | C/E (thousands of US$) | B/C* | DATI | C/E (thousands of US$) | B/C* |
| 50 days | 0.1  [0.1-0.4] | 9  [1.9-26] | 40  [30-40] | 0.2  [0.1-0.5] | 7.2  [1.4-21] | 50  [30-50] | 0.1  [0.1-0.2] | 8.3  [3-23] | 50  [30-50] | 0.2  [0.1-0.3] | 6.5  [2.3-19] | 60  [30-60] |
| 100 days | 0.3  [0.1-0.5] | 4.6  [1.4-13] | 90  [30-90] | 0.3  [0.2-0.7] | 3.9  [1-11] | 100  [30-100] | 0.2  [0.1-0.3] | 5.3  [2.5-13] | 70  [30-70] | 0.3  [0.2-0.4] | 4.4  [2-11] | 90  [30-90] |
| 150 days | 0.4  [0.2-0.6] | 3.5  [1.3-9] | 110  [40-110] | 0.4  [0.2-0.7] | 3.1  [1-8] | 130  [50-130] | 0.3  [0.2-0.3] | 4.6  [2.4-11] | 80  [40-80] | 0.3  [0.2-0.4] | 3.9  [1.9-9] | 100  [40-100] |

The COVID-19 vaccine was assumed to be 74% effective against infections and reducing infectivity, 95% effective against severity, and require 28 days from administration until the it becomes fully effective. 95% uncertainty ranges are presented in parentheses. DATI = deaths averted per thousand immunized; C/E = cost-effectiveness ratio; B/C = benefit-cost ratio; e = effectiveness of OPV vaccine against COVID-19. If e = 0%, costs outweighs benefits and should not be considered.

* Note the upper bound of the 95% uncertainty interval for B/C is the same as the mean. This is due to two reasons: first, all numbers are rounded to its nearest 10, so differences in the first digit is not shown. Second, the main result assumed high COVID-19 vaccine effectiveness, which were already at the upper end of the uncertainty ranges.

## **2.8 Derivation of VSL and standard sensitivity analyses for the benefit-cost ratios**

The GNI per capita (PPP, international $) in India in 2019 was $6,920 (World Bank 2021). We calculated the VSL estimates following the standard sensitivity analyses recommended by the benefit-cost analysis reference case (Robinson et al. 2019), and selected the lowest value ($388,000) for the main results.

Table A15. Range of VSL estimates and corresponding benefit-cost ratios for the COVID-19 scenario

e = effectiveness of OPV vaccine against COVID-19

t = days into wave both vaccines administered

d = days of COVID-19 vaccine delay after OPV administration

|  |  |  | Incremental benefit-cost ratio of adding OPV* | |
| --- | --- | --- | --- | --- |
|  | Derivation approach | VSL estimate | Simultaneous administration scenario | Delayed COVID-19 vaccine scenario |
| Standard sensitivity analysis 1 | VSL extrapolated from a US VSL of $9.4 million and US GNI per capita of $57900 (a VSL-to- GNI per capita ratio of 160), using an income elasticity of 1.5. if this approach yields a value of less than 20 times GNI per capita, then 20 times GNI per capita should be used instead | 388,000 | **e = 20%**  t = 25, B/C = 10  t = 50, B/C = 13  t = 100, B/C = 17  t = 200, B/C = 6  t = 300, B/C = 1  **e = 60%**  t = 25, B/C = 17  t = 50, B/C = 22  t = 100, B/C = 28  t = 200, B/C = 10  t = 300, B/C = 1 | **e = 20%**  **50 days into the wave that OPV is administered**  d = 50, B/C = 60  d = 100, B/C = 120  d = 150, B/C = 150  **100 days into the wave that OPV is administered**  d = 50, B/C = 60  d = 100, B/C = 100  d = 150, B/C = 110  **e = 60%**  **50 days into the wave that OPV is administered**  d = 50, B/C = 90  d = 100, B/C = 160  d = 150, B/C = 200  **100 days into the wave that OPV is administered**  d = 50, B/C = 90  d = 100, B/C = 130  d = 150, B/C = 150 |
| Standard sensitivity analysis 2 | VSL extrapolated from an OECD VSL-to-GNI per capita ratio of 100 to the target country using an income elasticity of 1.0 | 692,000 | **e = 20%**  t = 25, B/C = 17  t = 50, B/C = 23  t = 100, B/C = 30  t = 200, B/C = 11  t = 300, B/C = 1  **e = 60%**  t = 25, B/C = 30  t = 50, B/C = 40  t = 100, B/C = 49  t = 200, B/C = 17  t = 300, B/C = 2 | **e = 20%**  **50 days into the wave that OPV is administered**  d = 50, B/C = 110  d = 100, B/C = 210  d = 150, B/C = 270  **100 days into the wave that OPV is administered**  d = 50, B/C = 110  d = 100, B/C = 170  d = 150, B/C = 200  **e = 60%**  **50 days into the wave that OPV is administered**  d = 50, B/C = 170  d = 100, B/C = 290  d = 150, B/C = 360  **100 days into the wave that OPV is administered**  d = 50, B/C = 170  d = 100, B/C = 240  d = 150, B/C = 270 |
| Standard sensitivity analysis 3 | VSL extrapolated from a US VSL-to-GNI per capita ratio of 160 to the target country using an income elasticity of 1. | 1,107,000 | **e = 20%**  t = 25, B/C = 28  t = 50, B/C = 37  t = 100, B/C = 47  t = 200, B/C = 17  t = 300, B/C = 2  **e = 60%**  t = 25, B/C = 48  t = 50, B/C = 63  t = 100, B/C = 79  t = 200, B/C = 27  t = 300, B/C = 3 | **e = 20%**  **50 days into the wave that OPV is administered**  d = 50, B/C = 180  d = 100, B/C = 330  d = 150, B/C = 420  **100 days into the wave that OPV is administered**  d = 50, B/C = 180  d = 100, B/C = 270  d = 150, B/C = 310  **e = 60%**  **50 days into the wave that OPV is administered**  d = 50, B/C = 270  d = 100, B/C = 460  d = 150, B/C = 570  **100 days into the wave that OPV is administered**  d = 50, B/C = 270  d = 100, B/C = 380  d = 150, B/C = 430 |

* Comparing the scenarios with administering only the COVID-19 vaccine.

Note: if e = 0%, costs outweighs benefits and should not be considered.

## **2.9 Sensitivity analyses**

We conducted a comprehensive set of sensitivity analyses to determine the relative influence of varying individual parameters, including vaccine, epidemic, and cost parameters. The lower and upper ranges were chosen based on either the literature or realistic scenarios (Table A15). Changes in the incremental deaths averted per thousand immunized, cost-effectiveness and benefit-cost ratios are presented in Figures A5-6.

First, we observed that in both simultaneous and delayed coadministration scenarios, the model outputs are most sensitive to variations in the background epidemic parameters, such as R0, proportion of asymptomatic infections who become symptomatic, proportion of symptomatic infections that require hospitalization, and the ratio of infectiousness between asymptomatic and symptomatic infections. This suggests that the headline findings should not be generalized to other settings with different epidemic characteristics or other waves within the same country.

For the proportion of asymptomatic infections who become symptomatic, the main model assumes 7.4%, which we calculated by extrapolating from the national seroprevalence studies and supported by other studies (Kumar et al. 2021; Murhekar et al. 2021). Instead, if we reduce this to 3%, as reported by Murhekar et al. (2021), we would expect lower incremental benefits of OPV: approximately 50-60% reduction in deaths averted per thousand immunized (DATI), 50-200% increase in cost-effectiveness ratios, and 40-60% reduction in benefit-cost ratios (depending on the timing of coadministration). Instead, if we increase this proportion to 50% (i.e., half the asymptomatic infections become symptomatic), as reported in Chennai (Krishnasamy et al. 2021), we would expect greater incremental benefits of OPV: approximately 3-8 times DATI, 80-90% reduction in cost-effectiveness ratios, and 4-8 times greater benefit-cost ratios. In other words, a larger proportion of infections becoming symptomatic would lead to more deaths and therefore greater incremental benefit by co-administering OPV.

The relationship between model outcomes and R0 is more complex. We plotted the outcomes by different levels of R0 in Figures A7-8. In the simultaneous administration scenario, when co-administration occurs at day 25 of the wave, we observe a monotonic relationship between R0 and DATI and benefit-cost ratios: incremental benefits of OPV are greater with higher R0. For cost-effectiveness ratio, we see a sharp decline in the ratio as R0 increases from 1.0 to 1.5, followed by a gradual decline onwards. When co-administration occurs at day 50 of the wave, the largest OPV incremental benefit is round when R0 is between 1.5-2.0. When co-administration occurs at day 100 of the wave, the incremental benefits are relatively smaller and peak at lower levels of R0 (lower than R0 1.5). Furthermore, if the vaccines are administered later in the wave, and especially after the peak of the epidemic, the incremental gains from the interventions would be much smaller than if administered before the peak. In the delayed COVID-19 vaccine scenario, when R0 is less than 1.5, the relationship between R0 and incremental benefits depend on the length of the COVID-19 vaccine delay (*d*) and when OPV is administered (*t*). As expected, longer COVID-19 vaccine delays lead to greater incremental benefits from OPV. However, when R0 is greater than 1.5, incremental benefits of OPV are approximately the same regardless of the length of the COVID-19 vaccine delay. This is likely because with higher R0, the peak of the epidemic would occur earlier, and unless the COVID-19 vaccine is introduced much earlier (less than 100 days into the wave), the length of the delay would not make much difference in determining the incremental benefits of OPV.

Second, with respect to intervention parameters, the model outputs are most sensitive to OPV and COVID-19 vaccine effectiveness delay in the simultaneous administration scenario. This is because when the two vaccines are co-administered on the same day, OPV only provides incremental protection during the time gap required for the COVID-19 vaccine to become effective. Thus, having a shorter OPV effectiveness delay and/or longer COVID-19 vaccine effectiveness delay increases the incremental benefit of OPV. When the COVID-19 vaccine is delayed and administered 50-150 days after OPV, these two parameters become less critical because the time lag is larger than these effectiveness delays. Furthermore, specifically for economic evaluation results, we found the results to be sensitive to the OPV delivery cost input: a 50% increase in OPV delivery cost (from ~$1 to ~$1.5) would result in approximately 50% greater cost-effectiveness ratios and 33% lower benefit-cost ratios across all scenarios.

Finally, we note that most parameters related to the COVID-19 vaccine do not impact the results. This is expected, since these parameters are the same in both scenarios with and without OPV, therefore they are cancelled out when calculating the marginal benefits and costs between the two scenarios.

Table A16. One-way sensitivity analysis parameters

|  | Parameter | Value in main manuscript | Lower range | Upper range |
| --- | --- | --- | --- | --- |
| Vaccine | COVID-19 vaccine effectiveness against infections | 74% | 50% | 95% |
|  | COVID-19 vaccine effectiveness against infectivity | 74% | 50% | 95% |
|  | COVID-19 vaccine effectiveness against severity | 95% | 50% | 95% |
|  | COVID-19 vaccine effectiveness delay | 28 days | 7 days | 35 days |
|  | OPV effectiveness delay | 1 day | 1 day | 7 days |
| Epidemic | Basic reproduction number, R0 | 1.15 | 1.05 | 2.50 |
|  | Percent of infections who are symptomatic | 7.4% | 3% | 50% |
|  | Percent of symptomatic requiring hospitalization | 7.5% | 3% | 15% |
|  | Percent of hospitalized requiring intensive care | 10% | 5% | 20% |
|  | Percent of intensive care patients who recover | 85% | 50% | 95% |
|  | Infectiousness ratio between symptomatic and asymptomatic individuals | 0.75 | 0.1 | 1.0 |
| Cost | COVID-19 vaccine cost | $10 | $7.5 | $12.5 |
|  | COVID-19 vaccine delivery cost | $1.49 | 0.90 | $2.51 |
|  | OPV vaccine cost | $0.15 | $0.12 | $0.19 |
|  | OPV delivery cost | $0.96 | $0.56 | $1.56 |
|  | Vaccine wastage rate | 10% | 5% | 15% |

Figure A5. Tornado diagrams for one-way sensitivity analysis, simultaneous administration of both vaccines

t = days into wave vaccines administered; e = OPV effectiveness against COVID-19; C/E = cost-effectiveness ratio; B/C = benefit-cost ratio


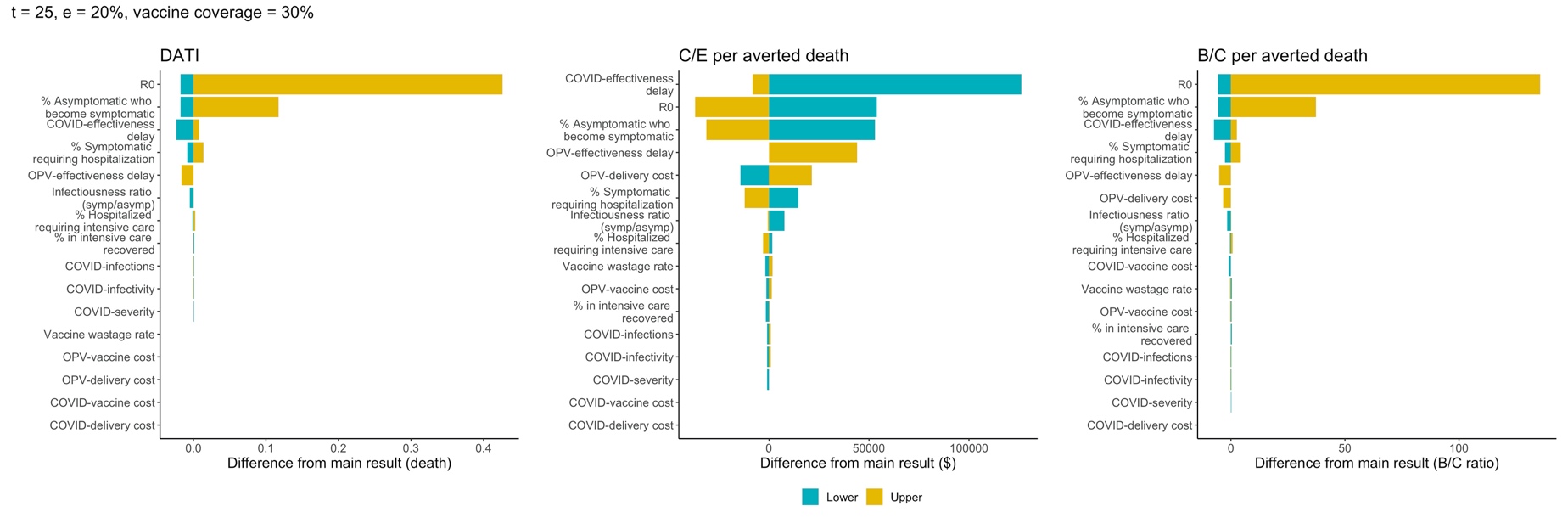


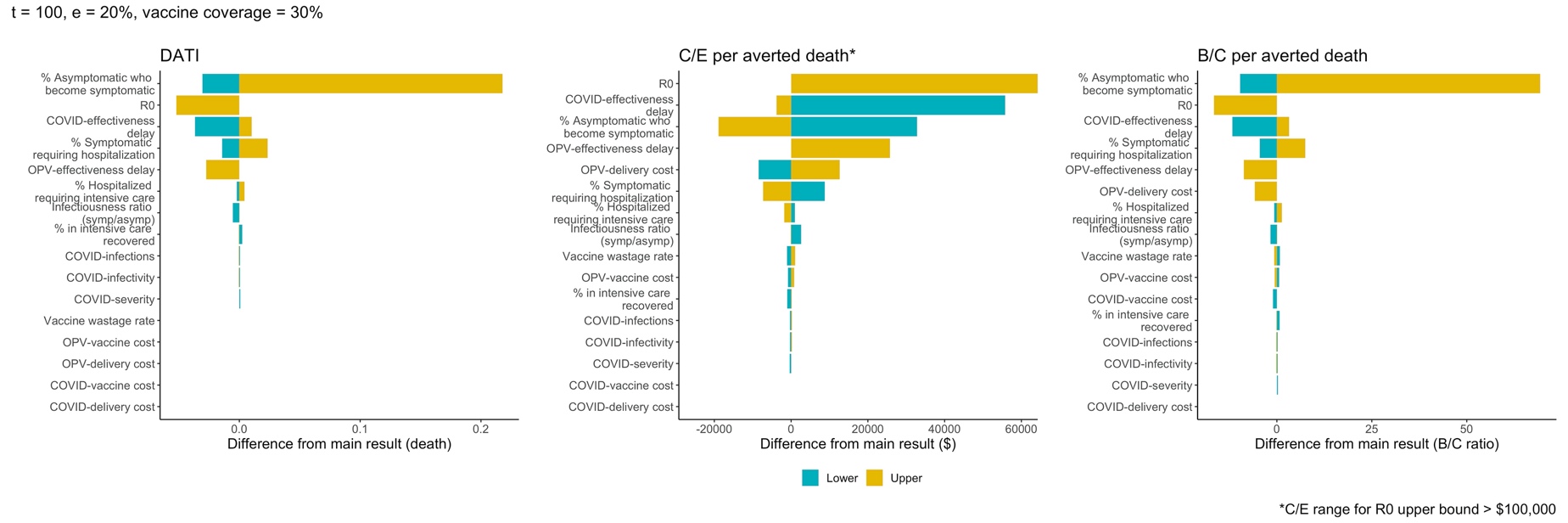


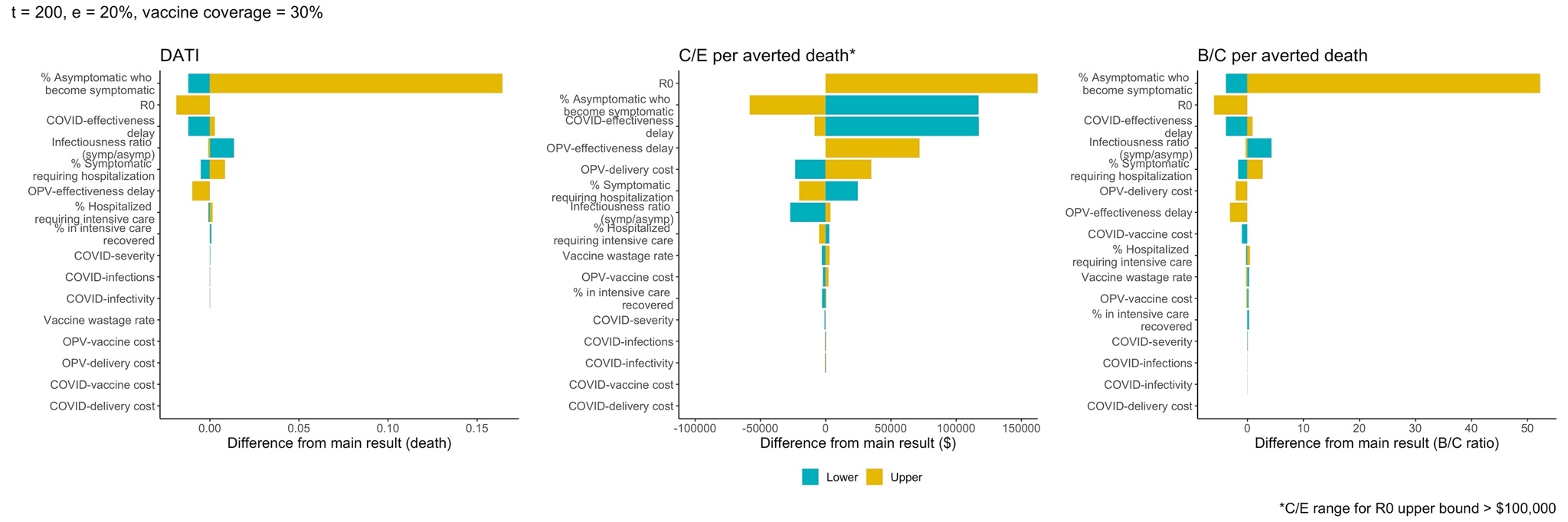

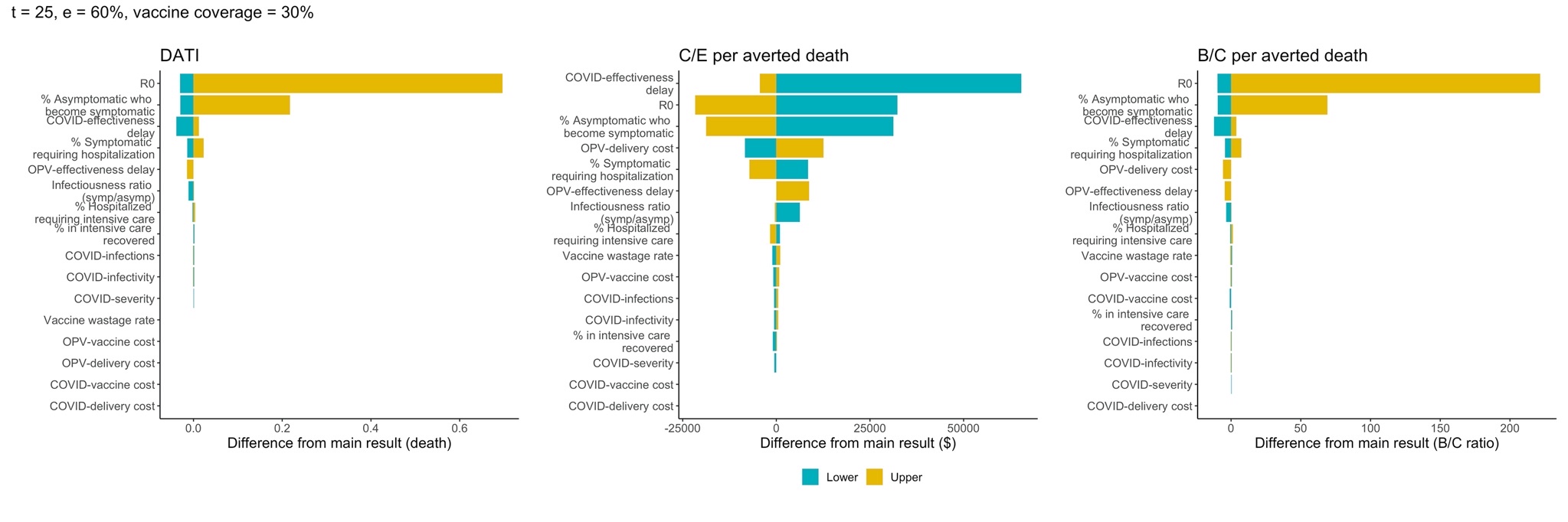

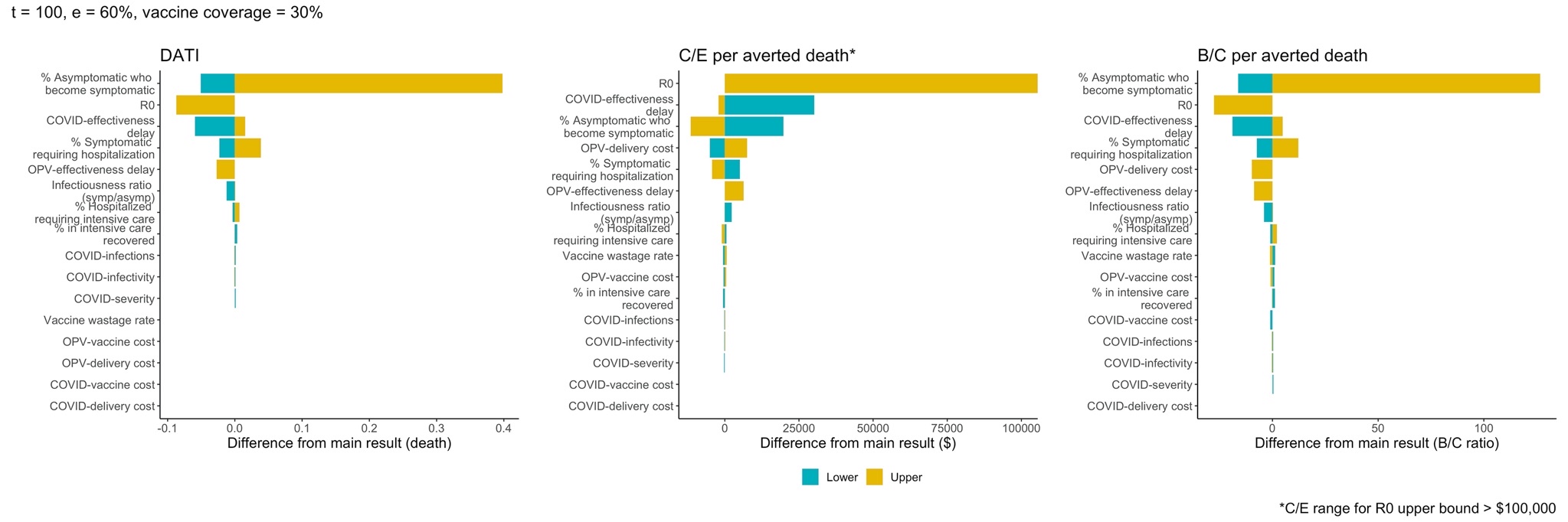

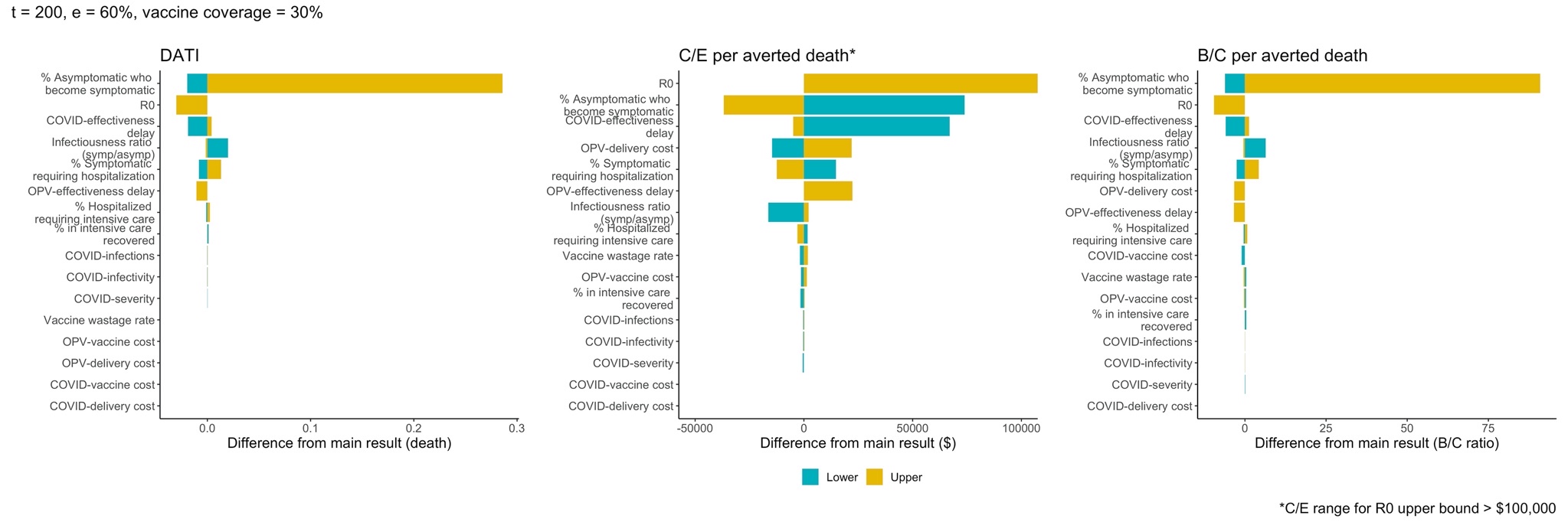


Figure A6. Tornado diagrams for one-way sensitivity analysis, co-administration with delayed COVID-19 vaccine


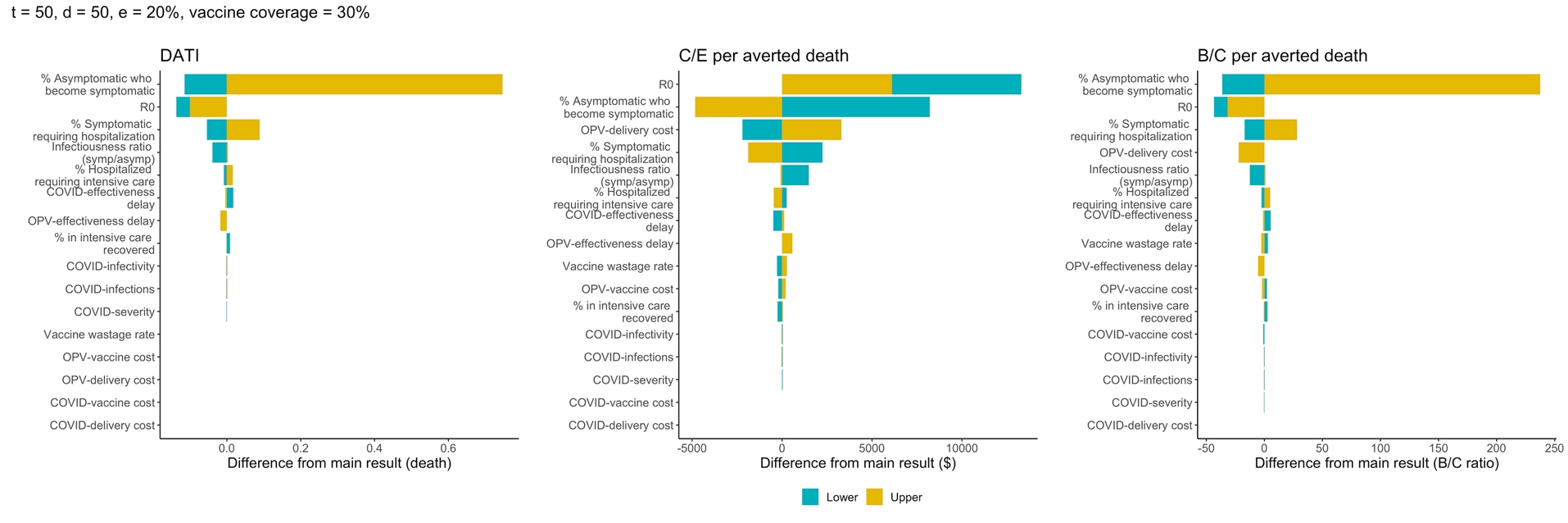


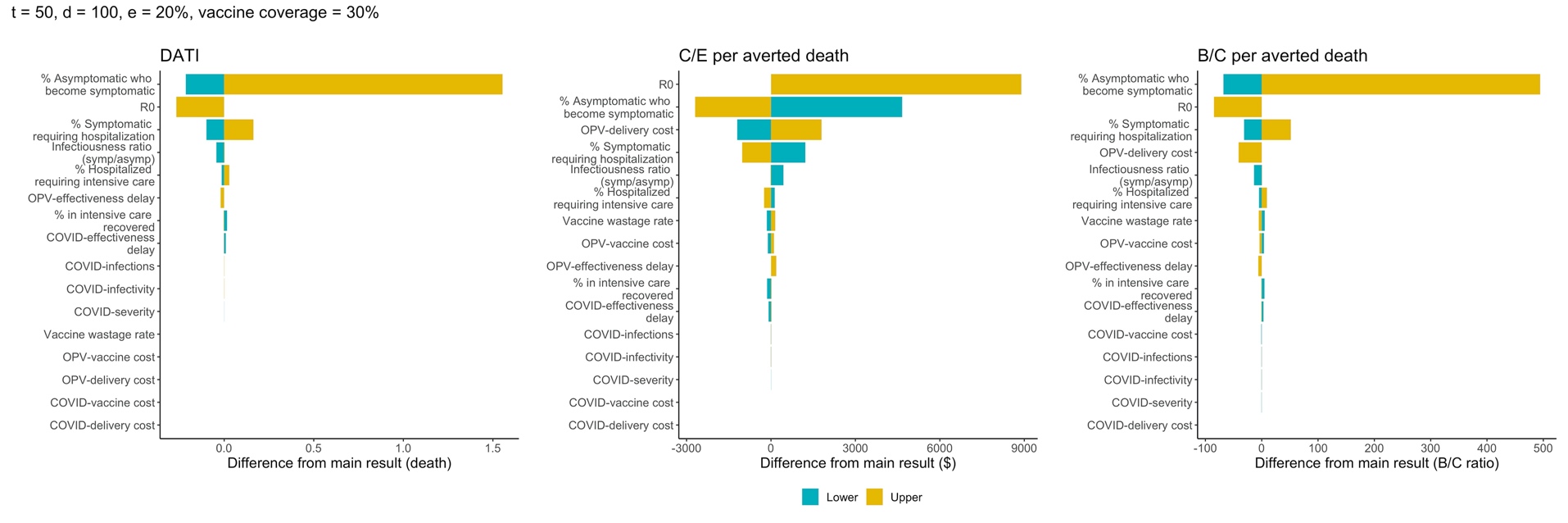


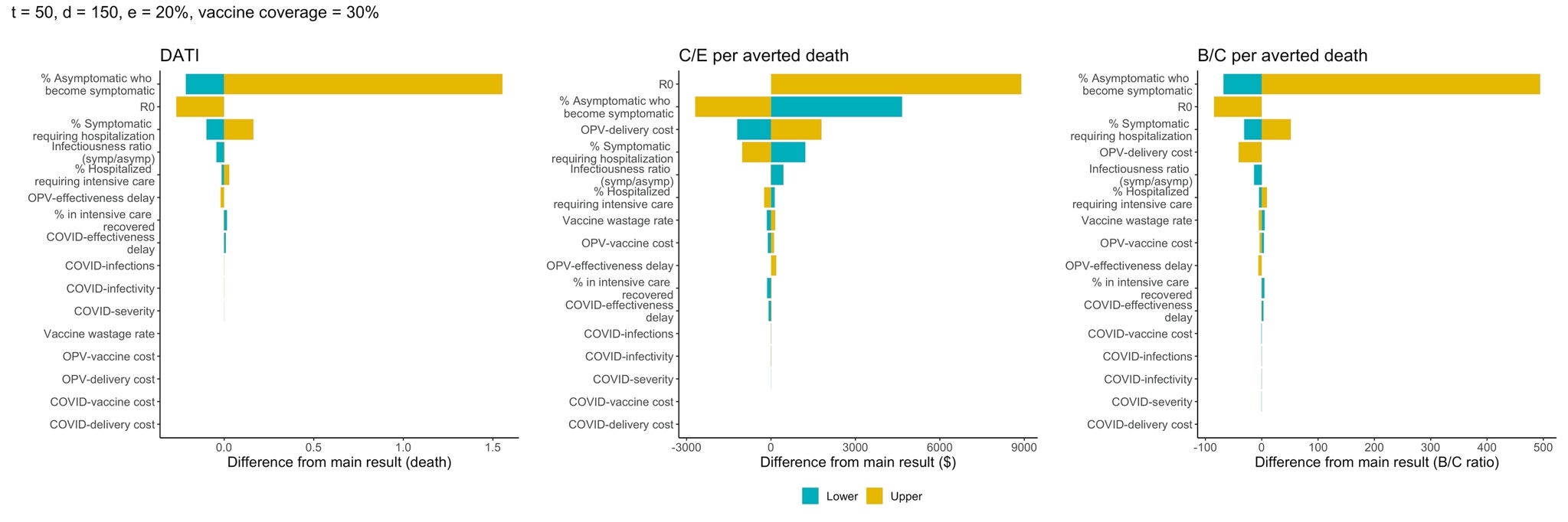

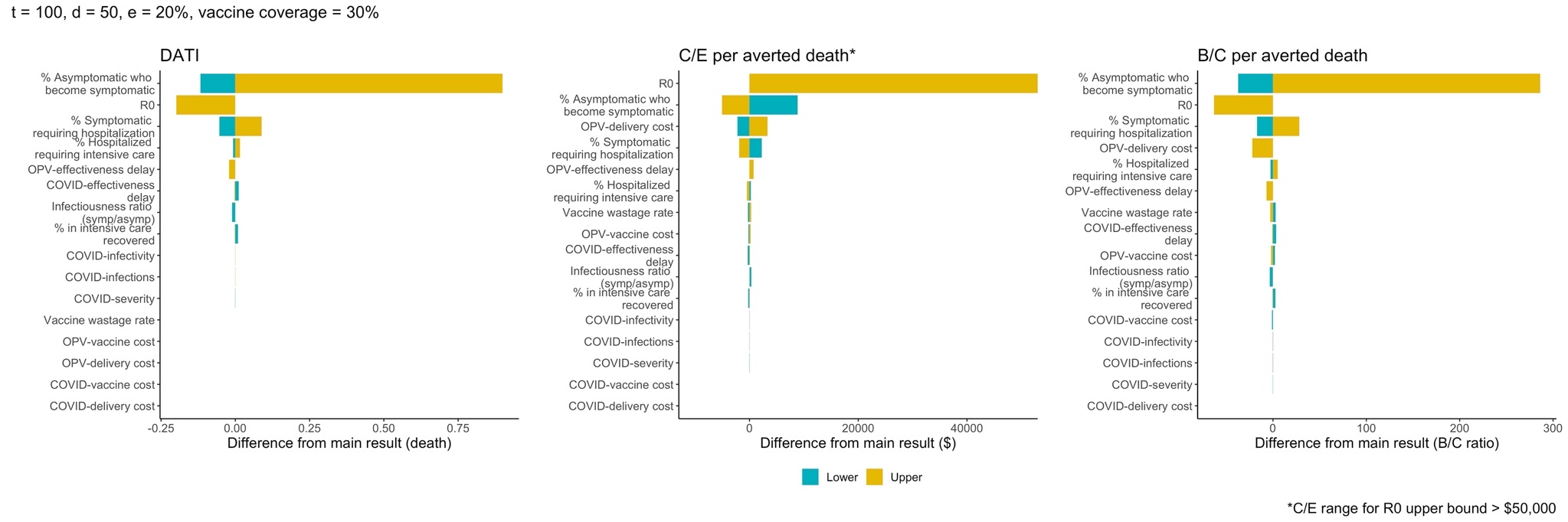

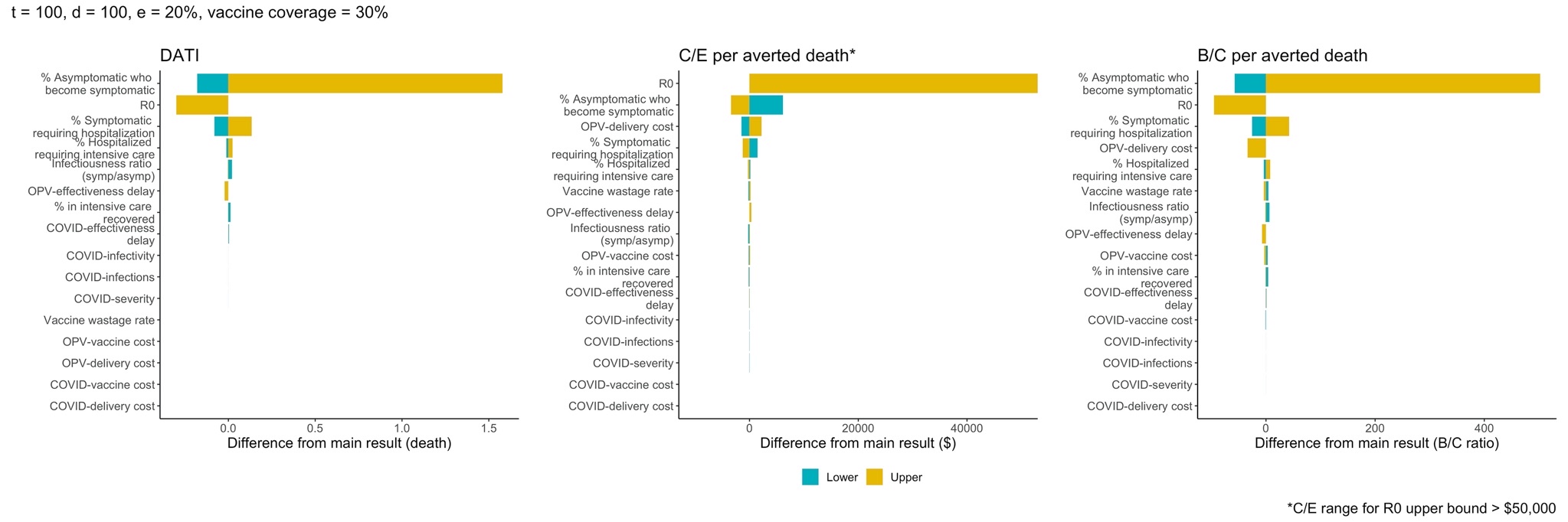

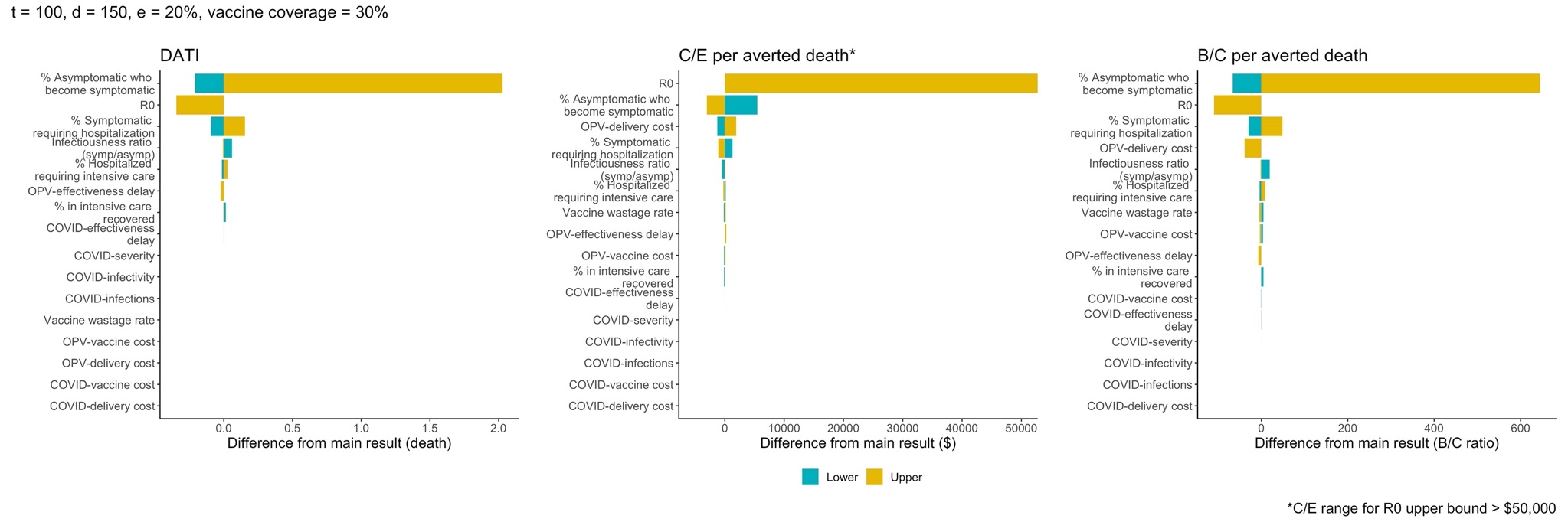


Figure A7. R0 and incremental benefits of OPV – simultaneous administration


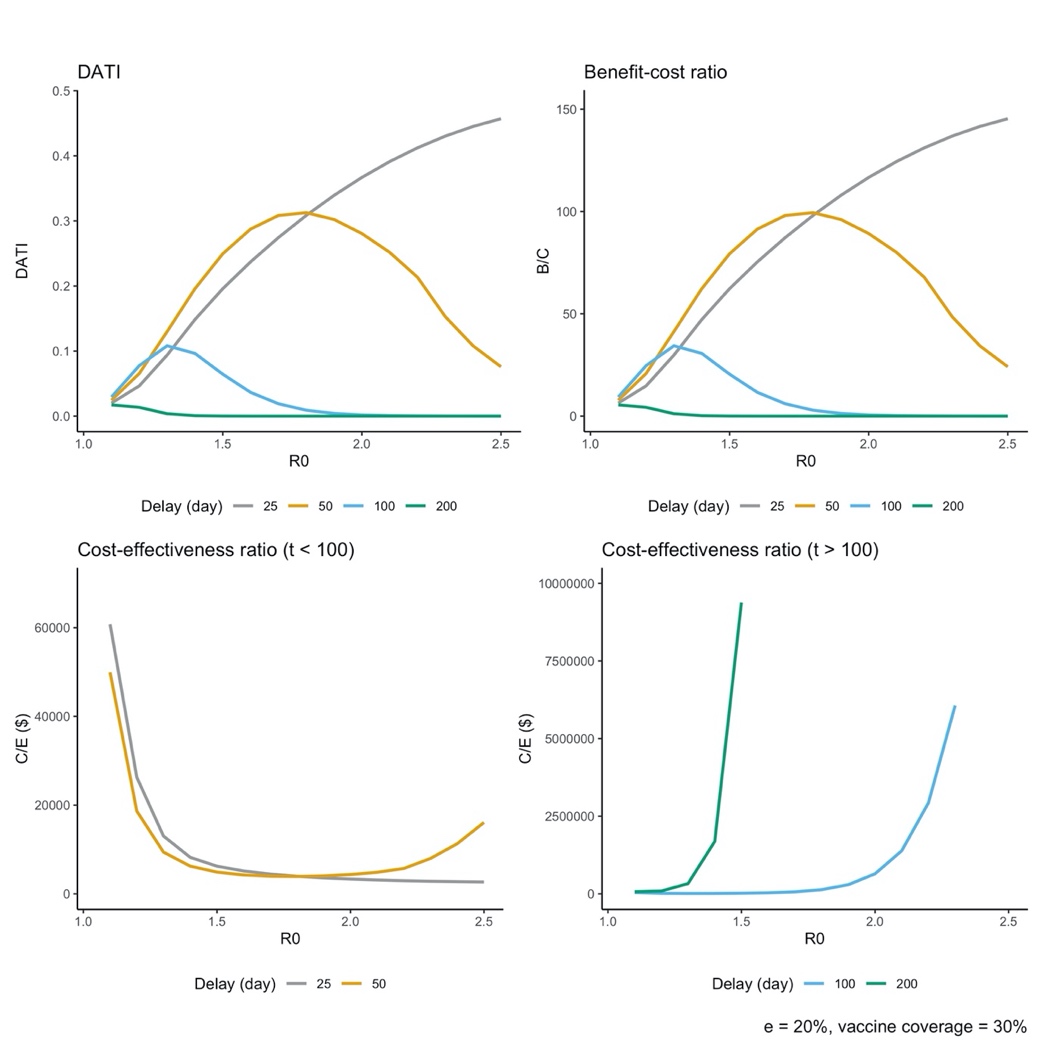


Both vaccines are administered on day *t*. DATI = deaths averted per 1000 immunized; e = effectiveness of OPV vaccine against COVID-19.

Figure A8. R0 and incremental benefits of OPV – delayed COVID-19 vaccine


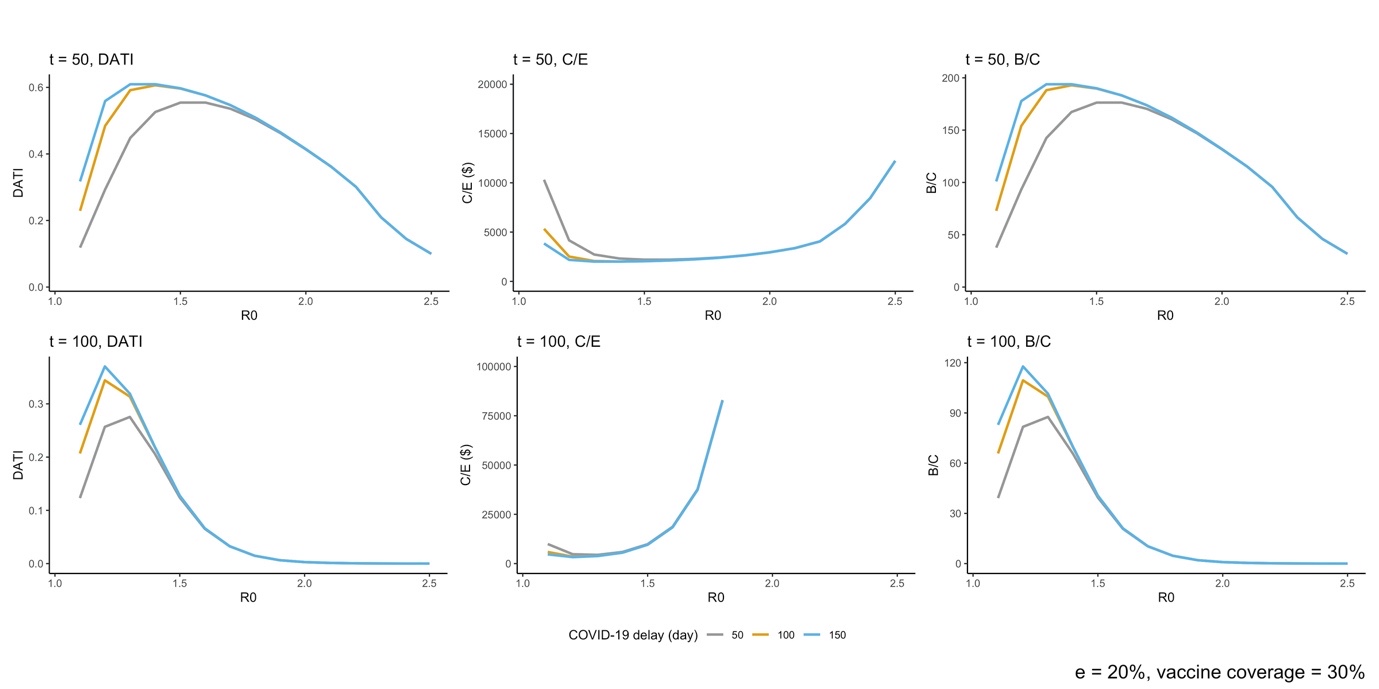


OPV is administered on day *t*, and the COVID-19 vaccine is administered *d* days after OPV. DATI = deaths averted per 1000 immunized; C/E = cost-effectiveness ratio; B/C = benefit-cost ratio; e = effectiveness of OPV vaccine against COVID-19.

## **Reference**

Bedi, Aneesha. 2020. ‘Andhra Sero Survey Finds Covid Exposure Higher in Urban Areas, over 92% Asymptomatic’. *ThePrint*. https://theprint.in/india/andhra-sero-survey-finds-over-92-covid-patients-asymptomatic-exposure-high-in-urban-areas/488314/ (May 6, 2021).

Cavailler, P et al. 2006. ‘Feasibility of a Mass Vaccination Campaign Using a Two-Dose Oral Cholera Vaccine in an Urban Cholera-Endemic Setting in Mozambique☆’. *Vaccine* 24(22): 4890–95.

Chumakov, M P et al. 1992. ‘Live Enterovirus Vaccines for Emergency Non-Specific Prevention of Mass Respiratory Diseases during Autumn-Winter Outbreaks of Influenza and Other Acute Respiratory Diseases’. *Journal of Microbiology, Epidemiology, and Immunology* 11–12: 37–40.

FDA. 2021. ‘Vaccines and Related Biological Products Advisory Committee Meeting February 26, 2021: Janssen Ad26.COV2.S Vaccine for the Prevention of COVID-19’. *FDA Briefing Document*. https://www.fda.gov/media/146217/download (April 3, 2021).

Fiedler, J. L. et al. 2014. ‘Child Health Week in Zambia: Costs, Efficiency, Coverage and a Reassessment of Need’. *Health Policy and Planning* 29(1): 12–29.

Fiedler, J. L, and T. Chuko. 2008. ‘The Cost of Child Health Days: A Case Study of Ethiopia’s Enhanced Outreach Strategy (EOS)’. *Health Policy and Planning* 23(4): 222–33.

Fisker, Ane, and Bandim Health Project. 2020. *Oral Polio Vaccine as Potential Protection Against COVID-19: A Cluster-Randomised Trial in Guinea-Bissau*. clinicaltrials.gov. Clinical trial registration. https://clinicaltrials.gov/ct2/show/NCT04445428 (April 11, 2021).

Griffiths, Ulla et al. 2021. ‘Costs of Delivering COVID-19 Vaccine in 92 AMC Countries’. : 27.

Jamison, Dean T. et al. 2013. ‘The Lancet Commissions’. *Lancet* 382: 1898–1955.

Jamison, Julian C. 2016. ‘Perceptions Regarding the Value of Life Before and After Birth’. *Reproductive System & Sexual Disorders* 05(04). https://www.omicsonline.org/open-access/perceptions-regarding-the-value-of-life-before-and-after-birth-2161-038X-1000195.php?aid=82198 (April 14, 2021).

Kar, Shantanu K. et al. 2014. ‘Mass Vaccination with a New, Less Expensive Oral Cholera Vaccine Using Public Health Infrastructure in India: The Odisha Model’ ed. Edward T. Ryan. *PLoS Neglected Tropical Diseases* 8(2): e2629.

Krishnasamy, Narayanasamy et al. 2021. ‘Clinical Outcomes among Asymptomatic or Mildly Symptomatic COVID-19 Patients in an Isolation Facility in Chennai, India’. *The American Journal of Tropical Medicine and Hygiene* 104(1): 85–90.

Kumar, Narendra et al. 2021. ‘Descriptive Epidemiology of SARS-CoV-2 Infection in Karnataka State, South India: Transmission Dynamics of Symptomatic vs. Asymptomatic Infections’. *EClinicalMedicine* 32. https://www.thelancet.com/journals/eclinm/article/PIIS2589-5370(20)30461-2/abstract (May 6, 2021).

MOST, USAID Micronutrient Program. 2004. ‘Cost Analysis of the National Vitamin A Supplementation Programs in Ghana, Nepal, and Zambia’. http://www.a2zproject.org/~a2zorg/pdf/GhanaNepalZambiaSythesis.pdf (April 26, 2021).

Murhekar, Manoj V. et al. 2020. ‘Prevalence of SARS-CoV-2 Infection in India: Findings from the National Serosurvey, May-June 2020’. *The Indian Journal of Medical Research* 152(1 & 2): 48–60.

Murhekar, Manoj V, Tarun Bhatnagar, Sriram Selvaraju, et al. 2021. ‘SARS-CoV-2 Antibody Seroprevalence in India, August–September, 2020: Findings from the Second Nationwide Household Serosurvey’. *The Lancet Global Health* 9(3): e257–66.

Murhekar, Manoj V., Tarun Bhatnagar, Jeromie Wesley Vivian Thangaraj, et al. 2021. ‘SARS-CoV-2 Sero-Prevalence among General Population and Healthcare Workers in India, December 2020 - January 2021’. *International Journal of Infectious Diseases*. https://www.sciencedirect.com/science/article/pii/S1201971221004422 (May 27, 2021).

Portnoy, Allison et al. 2015. ‘Costs of Vaccine Programs across 94 Low- and Middle-Income Countries’. *Vaccine* 33: A99–108.

———. 2020. ‘Producing Standardized Country-Level Immunization Delivery Unit Cost Estimates’. *PharmacoEconomics* 38(9): 995–1005.

Robinson, Lisa A et al. 2019. ‘Reference Case Guidelines for Benefit-Cost Analysis in Global Health and Development’. : 126.

Roser, Max, Hannah Ritchie, Esteban Ortiz-Ospina, and Joe Hasell. 2020. ‘Coronavirus Pandemic (COVID-19)’. *Our World in Data*. https://ourworldindata.org/coronavirus/country/india (April 15, 2021).

Seppälä, Elina et al. 2011. ‘Viral Interference Induced by Live Attenuated Virus Vaccine (OPV) Can Prevent Otitis Media’. *Vaccine* 29(47): 8615–18.

Thompson, Kimberly M., and Dominika A. Kalkowska. 2021. ‘Potential Future Use, Costs, and Value of Poliovirus Vaccines’. *Risk Analysis* 41(2): 349–63.

U.S. Department of Labor Bureau of Labor Statistic. 2021. ‘Consumer Price Index Data from 1913 to 2021’. https://www.usinflationcalculator.com/inflation/consumer-price-index-and-annual-percent-changes-from-1913-to-2008/ (March 10, 2021).

World Bank. 2021. ‘GNI per Capita, PPP (Current International $) | Data’. https://data.worldbank.org/indicator/NY.GNP.PCAP.PP.CD (February 24, 2021).
